# Framing the utility and potential pitfalls of relationship and identity DNA testing across United States immigration contexts

**DOI:** 10.1016/j.xhgg.2021.100060

**Published:** 2021-09-24

**Authors:** Diana Madden, Brianna A. Baker, Jennifer K. Wagner, Sara H. Katsanis

**Affiliations:** 1 Mary Ann & J. Milburn Smith Child Health Outcomes, Research, and Evaluation Center, Ann & Robert H. Lurie Children’s Hospital of Chicago, Chicago, IL 60611-2991, USA; 2 Stigma, Identity, and Intersectionality Research Lab, Teachers College, Columbia University, New York City, NY 10027, USA; 3 School of Engineering Design, Technology, and Professional Programs, Pennsylvania State University, University Park, PA 16802, USA; 4 Department of Pediatrics, Feinberg School of Medicine, Northwestern University, Chicago, IL 60611-2991, USA

**Keywords:** Immigration, ethics, ELSI, kinship, relationship DNA testing, identity DNA testing, family unity, vulnerable populations, STRs

## Abstract

Genetic information is increasingly used at US border entry points, but the use of DNA in immigration contexts is not new. DNA testing for verification of identity or relationships for visa and asylum petitions began in the 1980s. Long-standing applications demonstrate both the utility and pitfalls of DNA testing in immigration contexts. Some of these pitfalls are shared with health-related contexts of DNA testing, but the power of government officials to deny immigration benefits, separate families, or make accusations of fraud among a vulnerable population elevates the potential harms, including stigmatization, discrimination, and coerced consent. We conducted semi-structured interviews with professional stakeholders on their understandings of the process of DNA testing, opinions on the role of DNA testing in immigration, and experiences with DNA applications in immigration. From the 22 interviews, we sourced 21 case examples involving DNA testing and supplemented these with 10 case examples provided by the study team. The 31 case examples capture instances of DNA testing for relationship or identity across five immigration contexts. Using the case examples, we developed three overarching utilities and six overarching pitfalls of DNA testing that apply across these immigration contexts. Our framework allows long-standing applications of DNA testing in immigration to inform stakeholders’ approaches to applications in new contexts. As the use of DNA data in immigration contexts expands, its implementation should recognize the utility of DNA data to both migrants and government while guarding against pitfalls that could undermine the human rights and dignity of a vulnerable population.

## Teaser

The United States is expanding its use of relationship DNA testing in immigration contexts in volume and scope. Drawing on known case examples from interviews with professional stakeholders, we present a framework for understanding the utilities and pitfalls of relationship DNA testing across emergent use contexts.

## Introduction

The ethical and social implications of DNA relationship testing in the context of immigration are compounded by a combination of the vulnerabilities of migrant populations and the potential harms that might arise from testing.^1^, ^2^, ^3^, ^4^, ^5^ Many of the potential harms of DNA testing are shared across immigration and health-related contexts, including discrimination, stigmatization, privacy violations, revelation of sensitive information (such as misattributed parentage), and poorly informed or coerced consent. In immigration contexts, these risks are heightened by the power differential between those undergoing testing and those ordering the tests. While healthcare providers might be considered figures of authority, immigration agents or officials have the power to make decisions about families’ futures based on genetic information. The recent, rapid expansions of DNA testing for relationship verification in US immigration contexts—in both volume and purpose—demonstrate this power.

DNA data have been used to verify relationships for family-based immigration visas since a 1985 case in the United Kingdom.^6^ In the United States, DNA testing was first harnessed as evidence for family-based immigration visas in a legacy Immigration and Naturalization Services (INS) memo in 2000, which instructed that officers could suggest DNA testing in cases where other forms of documentation failed to verify a relationship.^7^ DNA testing has continued to be voluntary in most cases, with few exceptions (Table 1). Where DNA testing is voluntary, it might be requested by US Citizenship and Immigration Services (USCIS) after reviewing the evidence of the relationship provided; attorneys and/or clients also may choose to use DNA testing if they know there is little other documentation (e.g., birth certificate) of a relationship. Commercial relationship laboratories provide DNA testing for family-based immigration visas; currently, relationship testing laboratories must be accredited by the American Academy of Blood Banks (AABB) for results to be accepted in immigration and legal proceedings.^1^ Generally, only close relationships, such as parent-child or siblings, are tested. The genetic markers tested depend on the laboratory, but most use a set of 20–30 standardized, highly polymorphic short tandem repeat (STR) markers. The laboratory will issue a report indicating the likelihood of the tested relationship to the ordering party and the government agency seeking the results.^1^

**Table 1:** Contexts for relationship DNA testing in US immigration

Context	Background	Current status of DNA testing
Visa petition context: identity verification for visa or citizenship applications and petitions for non-citizen relatives	Relationship DNA testing was formally introduced to the US immigration system in 2000.^7^ In 2008, DNA testing was piloted among refugees for P-3 family reunification; fraud was reportedly revealed in East Africa,^8^ and the program was suspended.^9^ In 2012, the P-3 program resumed with a requirement for DNA testing,^9^ but it became defunct under the 2017 “Muslim ban.”^10^ Under the Obama administration, DNA testing was required to verify the parentage of petitioning Central American minors (CAM);^11^^,^^12^ the CAM program was closed during the Trump administration.^13^	Relationship DNA testing is conducted by AABB-accredited laboratories,^14^ most of which are commercial. Close relatives (e.g., parents, siblings) of petitioners might be asked to complete a DNA test in support of a relationship claim for, for instance, a Petition for Alien Relative (I-130) or Refugee/Asylee Relative Petition (I-730). Relationship DNA testing might also be used to verify identity and qualification for citizenship for an Application for Certification for Citizenship (N-600). DNA testing remains voluntary for most petitions but is required for the P-3 and CAM programs, to the extent that they are operational.
Unaccompanied youth context: relationship verification for placement with sponsors of unaccompanied migrant youth	Unaccompanied youth refers to children under 18 years of age who enter the United States without a parent or legal guardian or children who are separated upon entry from their adult caregiver.^15^ Since 2003, ORR has been responsible for the care and resettlement of UC, which includes determining the most suitable placement for a child.^16^^,^^17^	ORR states that “DNA matching is often used in the ORR UC program when documents are not available or unverifiable,”^18^ but the details of this use are unclear. In January 2021, ORR proposed revisions to two of the forms used to assess the suitability of a sponsor for a child; the proposed revisions include a change to form SVP-3/3 s that would allow sponsors “to voluntarily submit to a DNA test to prove they are biologically related to the child…in lieu of supporting paperwork.”^19^
Government separation context: verification of parent-child relationships following government-imposed family separation	The “zero-tolerance” policy was ended by executive order on June 20, 2018.^20^ Days later, a US District Judge ordered the reunification of migrant families within 14–30 days.^21^ HHS announced that it would use DNA tests to verify parent-child pairs in lieu of other forms of documentation to speed the process.^22^ Ongoing investigations have since revealed that the time frame and scale of separations were more extensive than previously thought.	As of June 2021, it is estimated by the Biden administration’s Interagency Task Force on the Reunification of Families that 2,127 children have not yet been reunified with their parent(s). The task force continues to identify instances of separation.^23^ Of the children thought to remain separated, it has not yet been possible to contact the parents, guardians, or attorneys of 368.^24^ These families could benefit from DNA-led reconnections outside of government control.^25^
Family verification context: verification of parent-child relationships at border entry points	In May 2019, the DHS conducted a pilot program at select border checkpoints over several months, motivated by reports of an increase in family unit fraud; the pilot was later extended from June to November 2019.^26^ Rapid DNA testing was used on site to verify claimed biological parent-child relationships in family units suspected of fraud.^20^	Over 5 months in 2019, the rapid DNA testing did not verify 24.7% of family units tested.^27^ The extent of the rapid DNA testing beyond 2019 is unknown; however, the 2020 biometrics proposed rule^27^ that was rescinded by the Biden administration^28^ proposed sustaining rapid DNA testing at the border.
Transnational missing context: comparison of FRSs to UHR samples for identification purposes in transnational missing persons cases	DNA from UHR is compared to FRSs to aid in identifications.^29^ Samples taken by US law enforcement can be uploaded to the CODIS, the federal DNA database of the United States; samples taken by NGOs are sent to private laboratories.^29^	Not all migrant remains are found, and those who are found might be buried or cremated before DNA is taken.^30^ Few families of missing migrants come forward to provide FRSs, possibly out of fear of law enforcement; others might approach an NGO to provide DNA. UHR samples and FRSs might be sent to different systems depending on the involvement of law enforcement and various NGOs in collection, resulting in silos of UHR and FRS data that cannot be compared.^31^

Abbreviations: P-3, Priority 3 refugee program; CAM, Central American Migrant Minors Program; AABB, American Association of Blood Banks; ORR, Office of Refugee Resettlement; UC, unaccompanied children; HHS, US Department of Health and Human Services; DHS, US Department of Homeland Security; UHR, unidentified human remains; FRS, family reference sample; CODIS, Combined DNA Index System; NGO, non-governmental organization.

Family-based DNA testing for the identification of people who die crossing the US-Mexico border is another long-standing use of DNA in an immigration context.^29^ Logistical, legal, and ethical challenges plague the DNA data sharing in missing migrants’ cases.^31^ Families may provide family reference samples (FRSs) for comparison to DNA samples from unidentified remains. For migrant families, families may choose to provide DNA FRSs to non-governmental organizations (NGOs) for comparison in a private database or to law enforcement for comparison to the federal DNA database, the Combined DNA Index System (CODIS). Any family member can provide an FRS, although ideally multiple close relatives, particularly maternal relatives, will provide samples. FRSs are typed for the 20 STRs commonly tested in forensic casework and that comprise the data in CODIS. FRSs in CODIS are not compared to the criminal evidence index, only to the unidentified remains index. While DNA identification of human remains can be successful, it is hampered by the fragmentation of unidentified human remains (UHR) and FRSs into different databases with policies preventing DNA data sharing.^2^

Beginning in 2018, new immigration contexts for the use of DNA testing gained the attention of both policymakers and the public. In April 2018, under the “zero-tolerance” immigration policy,^32^ migrant children were separated from their caregivers;^33^ soon after the practice was ended, the US Department of Health and Human Services (HHS) announced plans to use DNA to help reunify the separated families.^34^ Some of us authors tracked the extensive discussion by the media, policymakers, and the public of this potential DNA application.^35^ In May 2019, amid an increase in families arriving at the US-Mexico border, US Immigration and Customs Enforcement (ICE) began piloting the use of rapid DNA instruments at select border checkpoints to verify parentage claims of family unit pairs.^20^ Unlike DNA testing for family-based immigration visas, family verification testing at border sites is done on site using rapid DNA instruments. Currently, rapid DNA instruments test for STRs, and the policy indicates only parent-child relationships are tested.^26^ Families are selected for testing by government agents; testing is voluntary, but refusal might affect a family’s immigration status. In September 2020, DHS appeared to be poised to expand the scope of this rapid DNA testing with a Notice of Proposed Rule Making (NPRM) on Collection and Use of Biometrics by USCIS.^27^^,^^36^ The NPRM has since been retracted under the Biden administration.^28^ As 2021 progresses, the number of families and unaccompanied migrant youth arriving at the southern US border is rising steeply,^37^ and new bills have been introduced that suggest DNA testing to detect child trafficking.^38^ Several of us authors are part of efforts by the DNA Bridge consortium to develop trauma-informed processes and international, non-governmental structures to allow families to use DNA relationship testing to aid in locating and reunifying with their children.^25^

These recent expansions of DNA testing in both volume and scope, as well as continued attention from policymakers and the public, call for a framework for developing cross-context guidelines informed by long-standing uses of DNA data. Such guidelines should be grounded in an understanding of the utility of DNA tests as they pertain to measuring family relationships and the pitfalls that can emerge. We selected the terms “utility” and “pitfall” over familiar contrasting pairings such as pro/con, benefit/risk, or advantage/disadvantage, as each of these sets of terms implies a self-directed choice made by the person to pursue a DNA test. We borrow the term utility from the use of “clinical utility,” in that it captures the practical value of a test on health outcomes.^39^^,^^40^ The power dynamics, test type, and testing processes in the immigration context differ from a clinical context, so we have adapted the meaning of utility accordingly. Utility in an immigration context might include personal utility to an individual,^40^^,^^41^ institutional utility (i.e., to the immigration system), or societal utility. Our utility/pitfall framework encompasses both the benefits/risks to the person and the utilities/pitfalls of the DNA test within an immigration process. For instance, we can frame the utility of DNA test results as evidence in an immigration case to the petitioner and to the government, and we can frame the pitfall of the unexpected challenges or burdens arising from DNA testing processes in an immigration context.

To determine what the pitfalls and utilities of DNA relationship testing in immigration contexts might be, we conducted semi-structured interviews with key stakeholders involved with immigration and/or DNA testing in immigration contexts. We consider this framework over the course of the DNA testing process, including pre-testing processes such as requests for DNA testing and consent, sample collection and analysis, communication of results, and role of results in determining case outcomes. We outline the five currently relevant immigration contexts where DNA relationship testing might be used (Table 1) to provide clarity as to how the utility/pitfall framework applies across contexts. The recent expansion of DNA collection from migrant detainees for criminal justice purposes is an instance of the use of DNA data in an immigration context;^42^ in this context, however, DNA data are collected and held for criminal or missing persons investigations, not with the intention of conducting kinship analysis. Ancestry DNA testing also has been used in an immigration context to support or investigate nationality claims.^43^ We exclude both of these applications from our analysis, as our framework is tailored specifically to relationship DNA testing in immigration.^43^

Using a body of case examples sourced from our interviews and supplemented with case examples provided by the study team, we lay out a cross-context framework that captures the utilities and pitfalls of relationship DNA testing in immigration. As immigration to the United States continues to expand and new technologies, policies, and programs emerge, our framework based on factual case examples can be mapped onto new and shifting contexts to mitigate accumulation of harms.

## Subjects and methods

### Human subject protections

This study was conducted under Duke University Institutional Review Board (IRB) #2018-0510 and Lurie Children’s Hospital IRB #2019-2909. A consent information sheet was provided to participants prior to the interview, and verbal consent for audio recording was taken at the start of the interview. Permission for re-contact was also recorded. An NIH Certificate of Confidentiality covered the protocols and data collected from participants.

### Participant recruitment

We used information-oriented sampling to identify professional stakeholders involved with immigration processes who might know of or encounter DNA testing in their work, including immigration attorneys, NGO leadership and attorneys, technology company officials, academics, journalists, and representatives of government, law enforcement, and medicolegal agencies. We then compiled a further list of immigration attorneys from the American Immigration Lawyers Association website for random sampling, selecting for representation of geographical regions of the United States. We opted to speak with professional stakeholders, not immigrants or migrants and their families, because we anticipated that professional stakeholders would have a more varied experience of DNA testing in their fields. Additionally, we believed that the understanding and actions of the group of stakeholders we identified would have direct implications for immigrants or migrants and their families. This approach allowed us to explore how DNA testing plays out on the ground without interacting with potentially vulnerable groups. Recruitment emails were sent to candidate participants soliciting a reply of interest. We targeted 20–30 interviews with 2–3 participants from each stakeholder category, oversampling for immigration attorneys to gather diverse experiences.

### Semi-structured interviews

One semi-structured interview guide was used for all participants, regardless of profession. The guide focused on gathering data on the following: (1) knowledge of how DNA tests are used in immigration; (2) experience working with migrants, migrant families, and DNA testing; (3) basic comprehension of the purpose and process of DNA testing; and (4) opinions of the risks and benefits of DNA testing in immigration contexts. In addition, one Likert-scale question on the importance of DNA testing in immigration was initially included and later adapted into an open-ended question due to the struggle of participants to express their views via a numerical scale. Interviews were conducted in person or by phone between February and June 2019 and audio recorded to enable transcriptions.

### Interview coding

Interview transcripts were checked by two study team members, including one interviewer, for accuracy. Transcripts were then coded to capture: (1) applications of DNA testing in immigration contexts, (2) scope of participant experiences and knowledge of DNA testing, and (3) participant perspectives on general benefits and risks of DNA testing in immigration. Interviews were further coded for any mention of specific cases involving DNA testing, descriptions of the types of situations where DNA testing is used, and descriptions of DNA testing processes. Each case, use instance, and process description was summarized from participant statements and collated into a spreadsheet.

Coders then generated a set of pitfalls that emerged as themes across all interviews. While the pitfalls emerged from descriptions of cases, use instances, and processes, descriptions of specific case examples best illustrated these pitfalls. Case examples were then coded with additional categories: case type, immigration benefit sought, relevant biological/legal/social relationships, case status, number of people involved and their location(s), details of DNA test type if any, who requested testing, who was tested, instance of fraud (yes/no [Y/N]), and instance of misattributed parentage (Y/N).

### Development of case examples

Participant descriptions of cases with a high level of contextual detail were selected for development into case examples. Our aim was to capture the broadest possible range of actual uses of DNA testing in an immigration context. The summaries of the case examples from the initial coding process were refined into narrative form (Supplemental notes). The coding of the case type was used to assign each case example to one of the five immigration contexts (Table 1). Case examples were grouped by immigration context and titled with a brief description of the exemplified pitfall(s) or utility(ies). Participants who provided certain cases that were missing key details needed to develop the narrative consistently across examples were re-contacted for additional information, and their responses were incorporated into the specific case example narrative.

The case example narratives were reviewed by two coders to check for accuracy and clarity. Interview transcripts were referenced where necessary to verify that the participant’s meaning and the level of detail provided were preserved in the narrative. A third coder read the final case examples for clarity and accessibility to audiences of diverse expertise.

Parallel to the development of case examples from interviews, study team members were asked to share cases within the five immigration contexts that came to their attention. Most of these cases were already in the public domain in academic, governmental, or media reports; these were anonymized, coded, and developed into narratives.

### Development of pitfalls and utilities

Case examples were grouped according to similarities between the pitfalls and/or utilities of DNA testing that they demonstrated. Case examples could belong to more than one group. A summary statement of the utility(ies) or pitfall(s) demonstrated by each group was crafted to capture the elements that applied across cases and immigration contexts. Coders reviewed the applicability of each pitfall to each immigration context. We requested the input of the study team on the immigration contexts framework and integrated their feedback. Finally, two independent coders re-coded the case examples to ensure that they fit within the overarching framework.

## Results

### Participants

A total of 181 professionals were identified for potential contact: 125 immigration attorneys, 33 NGO representatives, nine government or law enforcement officials, nine academics, and five technology company representatives. Invitation and follow-up invitation emails were sent to all but six of these stakeholders. Of the 175 invitations, 28 responded (response rate of 16%), and 23 (13%) agreed to interviews. The 23 participants included 13 immigration attorneys, five NGO representatives, two academics, and three technology company representatives; participants are represented here via codes that reflect their profession (Table 1). TC08 and TC09 were interviewed together. One participant declined audio recording; all agreed to re-contact.

### Participant experience with DNA testing in immigration contexts

Participants’ interactions with immigrants and/or migrants and experience with DNA testing in immigration contexts varied by profession and in some cases between participants. Overall, the experiences of the participants slanted toward family-based applications for US visas (or petitions by refugees or asylees). The companies that the three technology company representatives (TC07, TC08, TC09) worked with specialized in immigration-related DNA testing. Both of the academics (AC03, AC05) and the three NGO directors (NG02, NG06, NG14) had limited direct interaction with immigrants and/or migrants but engaged as supervisors or through their professional work with multiple contexts where DNA testing might occur. The two NGO attorneys (NG15, NG21) worked primarily on family-based applications for refugees and asylees and sponsorships for unaccompanied minor children. Most of the immigration attorneys’ encounters with DNA testing involved visa or citizenship applications and petitions for noncitizen relatives.

Of the 11 participants who gave an explicit characterization of how often they encountered DNA testing in their work with immigrants and/or migrants, 10 stated that they encountered DNA testing infrequently; for instance, IA01 summarized, “maybe I’ll have one [instance of DNA testing], maybe two a year.” Only one participant, IA10, reported frequent DNA testing, stating, “Yes, actually, I—we see it quite a bit and in fact I feel like the practice of requesting DNA test—tests has really gone up in the last 10 years or so where you can expect it on, you know, a good percentage of the cases.”

Despite overall infrequent experience of DNA testing, participants emphasized its importance in immigration contexts. Participants IA01, NG02, AC05, and IA04 struggled to assign a number to the importance of DNA testing in immigration on a Likert scale (with “5” being very important). IA01 captured the tone of the ambiguity, saying, “No, I mean, I think on the cases where it is used, it’s probably a five because it's really important in those specific cases to be able to have some type of biological match. But it’s just, it’s not something that we commonly use.” In lieu of the Likert-scale question, the remaining participants were asked how much weight is given to DNA testing in immigration contexts, which prompted wider-ranging considerations of the utility of DNA to different stakeholders (attorneys, clients, or the government). Participants particularly emphasized the ability of positive or negative results of DNA testing to determine the outcome of a case: for instance, IA10 stated, “Yeah, I mean, well, it’s helpful if it’s a match. And it’s incredibly unhelpful if it’s not a match. You know? …if the government is requesting a DNA test, um, whether it’s positive or negative will be the determining factor in the case.” NG02 emphasized the value placed by the government on DNA test results as “clear objective evidence” of a relationship.

### Case examples

Coding of the interviews yielded 45 cases, 42 use instances, and 19 descriptions of processes. Of the 45 cases, 21 were developed into case example narratives (Table 2). The 24 cases not selected for narrative development were similar in content to cases for which more detail was provided. An additional 10 cases were sourced from the study team, of which seven are in the public domain. The full catalog of case examples, referenced here by case number, is available as Supplemental notes. No case examples were sourced from nine of the participants. Four case examples were sourced from NG06, with one to two case examples sourced from each of the remaining participants. Twenty-two of the 31 total cases fell under the visa petition context, including all but three of the case examples sourced from participants. The transnational missing context had six case examples, all of which were supplied by the study team.

**Table 2:** Characteristics of case example sources

Participant or source	Profession	Gender	Visa petition context	Unaccompanied youth context	Government separation context	Family verification context	Transnational missing context
IA01	immigration attorney	M	cases 8, 9				
IA04	immigration attorney	F	case 2				
IA10	immigration attorney	F					
IA11	immigration attorney	M	case 4				
IA12	immigration attorney	M					
IA13	immigration attorney	M	case 18				
IA16	immigration attorney	F		case 20			
IA17	immigration attorney	F	cases 13, 16				
IA18	immigration attorney	F	case 7				
IA19	immigration attorney	M	case 1				
IA20	immigration attorney	M					
IA22	immigration attorney	F					
IA23	immigration attorney	F	case 14				
NG02	NGO director	M			case 21		
NG06	NGO policy director	F	case 15	case 12			
NG14	NGO director	F					
NG15	NGO attorney	F	cases 11, 17				
NG21	NGO attorney	F	cases 3, 10, 19				
AC03	academic	M					
AC05	academic	F					
TC07	technology company representative	M	case 6				
TC08 & TC09	technology company representative	M, F	case 5				
Study team		N/A	cases 22, 23, 24			case 25	cases 26–31

See Table 1 for context descriptions. M, male; F, female; N/A, not applicable.

### Utilities and pitfalls of DNA testing in immigration contexts

We identified three overarching utilities and six overarching pitfalls that apply to DNA testing for relationship verification across the five immigration contexts. The three utilities are: (A) DNA testing can provide documentation of genetic relationships when other forms of documentation are unavailable, inaccurate, or insufficient to meet the burden of evidence; (B) DNA testing can disprove or detect fraud when claimed relationships or identity are in question; and (C) DNA testing requests or requirements can deter intentionally fraudulent misrepresentations of relationships or clarify misunderstandings around the kinds of relationships that qualify for immigration benefits. The six pitfalls are: (A) Family is not defined by genetic relationships alone; kinship terms do not correspond to biological (or genetic) relationships in the same way across languages and cultures; (B) kinship analysis can reveal sensitive information; (C) collection, processing, and comparison of DNA samples from multiple individuals can carry logistical, temporal, geographical, and financial burdens; (D) DNA from the appropriate individuals to test a relationship is not always available; (E) the appropriate technology and/or infrastructure to test a relationship is not always available; and (F) the government might not collect samples or request or apply DNA testing results uniformly in decision-making processes. Case examples often demonstrated both utilities and pitfalls. Of the 31 cases, 11 were coded for multiple pitfalls, and 12 were coded for both a utility and at least one pitfall. Table 3 shows case examples by utility and immigration context, and Table 4 shows case examples by pitfalls and immigration context.

**Table 3:** Utilities of relationship and identity DNA testing across US immigration contexts

	Visa petition context	Unaccompanied youth context	Government separation context	Family verification context	Transnational missing context
No utility stated or interpreted	cases 9–11 12, 15, 19, 22^a^	no case examples from study team; case 20	no case examples from study team; case 21	no case examples	no case examples from interviews; cases 30,^a^ 31^a^
Utility A: DNA testing can provide documentation of genetic relationships and identity when other forms of documentation are unavailable, inaccurate, or insufficient to meet the burden of evidence.	cases 1–3, 13, 14, 16–18, 24^a^	no case examples	no case examples	no case examples	no case examples from interviews; cases 26–29^a^
Utility B: DNA testing can disprove or detect fraud when claimed relationships or identity are in question.	cases 4–6, 08, 23^a^	no case examples	no case examples	no case examples	no case examples
Utility C: DNA testing requests or requirements can deter intentionally fraudulent misrepresentations of relationships or clarify misunderstandings around the kinds of relationships that qualify for immigration benefits.	no case examples from study team; case 7	no case examples	no case examples	no case examples from interviews; case 25^a^	no case examples

See Table 1 for context descriptions.

a Case examples sourced from the study team, not from interviews.

**Table 4:** Pitfalls of relationship and identity DNA testing across US immigration contexts

	Visa petition context	Unaccompanied youth context	Government separation context	Family verification context	Transnational missing context
No pitfalls stated or interpreted	cases 1–6	no case examples	no case examples	no case examples from interviews; case 25^a^	no case examples from interviews; case 26^a^
Pitfall A: family is not defined by genetic relationships alone; kinship terms do not correspond to biological (or genetic) relationships in the same way across languages and cultures	cases 7, 22^a^	no case examples	no case examples	no case examples	no case examples
Pitfall B: kinship analysis can reveal sensitive information	cases 8, 9, 10, 11, 12, 22^a^	no case examples	no case examples	no case examples	no case examples from interviews; case 27^a^
Pitfall C: collection, processing, and comparison of DNA samples from multiple individuals can carry logistical, temporal, geographical, and financial burdens	cases 13, 14, 15, 23,^a^ 24^a^	no case examples	case 21	no case examples	no case examples from interviews; cases 27,^a^ 28^a^
Pitfall D: DNA from the appropriate individuals to test a relationship is not always available	case 14	no case examples	no case examples	no case examples	no case examples from interviews; cases 28,^a^ 29^a^
Pitfall E: the appropriate technology and/or infrastructure to test a relationship is not always available	cases 14, 15, 16	no case examples	no case examples	no case examples	no case examples from interviews; cases 28,^a^ 29,^a^ 30^a^
Pitfall F: the government might not collect samples or request or apply DNA testing results uniformly in decision-making processes	cases 12, 16, 17, 18, 19, 22,^a^ 23,^a^ 24^a^	case 20	no case examples	no case examples	no case examples from interviews; cases 27,^a^ 29,^a^ 30,^a^ 31^a^

See Table 1 for context descriptions.

a Case examples sourced from the study team, not from interviews.

#### Utility A: DNA testing can provide documentation of genetic relationships when other forms of documentation are unavailable, inaccurate, or insufficient to meet the burden of evidence

Participants named lack of documentation as a primary reason DNA testing is used for visa or citizenship applications and petitions for noncitizen relatives; often, DNA testing ultimately supported access to an immigration benefit by providing documentation of relationships. Participants often described challenges presented by birth certificates. In case 01 and case 18, DNA testing provided genetic documentation of a father-child relationship where the father was not listed on the birth certificate. In case 02, the grandparents had chosen to be listed as the parents on the birth certificate of their grandchild, whom their young daughter had out of wedlock; DNA testing provided genetic documentation of a mother-child relationship for their daughter and grandchild’s visa petition. In case 13, the birth certificate correctly listed the relevant father-child relationship, but the certificate’s authenticity was questioned; DNA testing provided documentation. In case 14, a client had been deported several times despite a birth certificate naming a US citizen as his father; the client and attorney hoped that DNA testing would demonstrate the paternal relationship to support a claim to citizenship, although testing had not been completed at the time of the interview.

Participants also described cases where the absence of a history of interactions or a caregiving relationship was the primary barrier to the desired immigration benefit. For example, describing an as-yet-unresolved case (case 17), NG15 questioned the potential usefulness of DNA testing as documentation to support the reunification of a father and child estranged due to war, given the lack of a caregiving relationship. In case 18, DNA documentation initially was considered insufficient to support the reunification of a father and child with no history of a caregiving relationship, but the initial denial was ultimately overturned on the basis of the genetic relationship.

Documentation or clues to identify and connect transnational missing persons to families also can be scarce, making DNA testing a valuable tool. DNA testing in combination with other evidence in case 26 allowed for the identification of a person whose family had been searching for her. But DNA testing does not always provide resolution. In cases 28 and 29, forensic anthropologists submitted DNA samples from UHR for upload to CODIS, but to date no FRSs have matched.

#### Utility B: DNA testing can disprove or detect fraud when claimed relationships or identity are in question

In some case examples, government suspicion was directed at those seeking an immigration benefit, and DNA testing helped remove suspicion. In case 04, a DNA test was ordered by a client and his attorney to provide evidence of the client’s identity, relieving the government’s suspicion that he was not eligible for naturalization. In case 23, a man who was born to two US citizens and adopted abroad was in danger of being deported from the United States; he hoped DNA testing, in conjunction with other evidence, would demonstrate his claim to US citizenship. In case 08, it was the client and her attorney who used a DNA test to verify a claim. The client had a child whom she believed had died as an infant. Years later, an adult approached her claiming to be the child and seeking to join her in the United States. DNA testing was used to verify the relationship prior to submitting a petition for the child. While it alleviated the concerns of the attorney and client, the DNA test opened the client up to government suspicion. The question arose as to whether she had committed fraud in the past by not disclosing the existence of her child. DNA testing can help the government detect fraud, as in case 05. In this case, when the brother and sister samples submitted to a laboratory both profiled as male, and re-sampled as male, the unusual finding triggered an investigation; the investigation indicated that the same samples, known to be genetically related, were being intentionally submitted in place of actual samples of the family members. In case 06, the State Department reviewed submissions for different individuals from the same country and found that the same profile had been used for multiple cases.

#### Utility C: DNA testing requests or requirements can deter intentionally fraudulent misrepresentations of relationships or clarify misunderstandings around the kinds of relationships that qualify for immigration benefits

In case 7, IA18 claimed to have experienced cases where a request for DNA testing revealed that clients’ social, legal, and genetic relationships did not align with how they had been presented in the petition. They described conversations with various clients, for instance, “Often it’s, ‘Well I raised, you know, Susan since she was an infant. Her mother is my sister and couldn’t take care of … in fact, she’s my niece.’” IA18 indicated that clients sometimes seemed to have intentionally concealed the true relationship and sometimes to have misunderstood the documentation and relationship required to support their petitions. IA12 described similar conversations, stating, “I certainly see those cases, in which case I’ve had to advise clients, you know, because that isn’t your biological daughter…, and you aren’t actually married to the mother, or just whatever circumstances, …you know, we’re not able to move forward with that petition.”

#### Pitfall A: Family is not defined by genetic relationships alone; kinship terms do not correspond to biological (or genetic) relationships in the same way across languages and cultures

NG21 captured this pitfall when describing the risks of DNA testing:

Emotionally I think there are [risks]. I think—I’m not a scientist, but it seems like a pretty straightforward process as far as swabbing the inside of your cheek—I think what is maybe the problem is our immigration laws are written from a very Western, white perspective of what a family is.

A comment from NG02 further explained the appeal and risks of DNA evidence:

You know, all this indica [*sic*] of, of parental relationship[s] and it’s just, it’s just easier for the government to have this clear objective evidence. Now I do admit that the government is always concerned about fraud…. But when they create a rule that they think is, well this is good, this, this will prove it, they’re—they’re ignoring the consequences of that rule—right, and the hardship. It could be economic hardship, it could be stress, it could be error in the testing. It could be that the child is not the biological child of the parent. So, um, I don’t know why the law would privilege biology over behavior.

NG15 also noted that when genetic relationships do not match claimed relationships, petitioners might have their application denied or be labeled fraudulent. Two case examples demonstrate particularly well the extent family structures might not align with policy. IA18’s conversations with petitioners in case 7, described above, indicated that there was confusion around relationship terms for some families. Case 22 highlighted a family with caregiving, genetic, and legal relationships that nevertheless faced barriers to immigration; a same-sex married couple, one a US citizen and one a foreign citizen, applied for US citizenship for their children born via surrogacy. Each child was genetically related to one parent; only the child genetically related to the US citizen was granted citizenship. This case was later reversed in courts, granting citizenship to both children.

#### Pitfall B: Kinship analysis can reveal sensitive information

DNA testing can reveal unknown or unexpected information about relationships (e.g., misattributed parentage). Participants also illustrated how conversations leading up to DNA testing could reveal sensitive information about family relationships. The effect of DNA test results on the immigration process, introduced under pitfall A above, and lack of preparation on the part of professionals to communicate results, might compound any trauma from the revelation of sensitive information. Case 9, in which a man wished to bring the child he had with a woman abroad to the United States, demonstrated all three of these aspects of this pitfall. A man revealed to his wife that he had a child abroad with another woman and filed a petition for the child. During the application process, DNA testing was requested and unexpectedly revealed that he was not the genetic father. IA01 described the father’s reaction as “dumbfounded.” With no genetic or legal connection to the child, the petition was denied; outside of undergoing a legal adoption process, the man was left with no options to bring the child to the United States. IA01 summarized the effect of DNA testing on cases like this one:

If the DNA turns out that, hey, that’s not your child or that’s not your parent or that’s not your brother or sister, then, then that’s really it. Then that person, regardless of what kind of emotional connection you have with that individual or however much time you spent, that person is just a random person in your life and they’re not necessarily eligible for benefits.

Misattributed paternity also ended the petition under the Central American Migrant Minors Program of the father in case 10. NG21 alluded to the challenge of communicating results of nonpaternity to a man who believed he was the genetic father: “[The attorney] was one of my colleagues that I shared an office with. And, yeah, she was definitely not prepared to give him those results.” Case 11 demonstrated another type of trauma that can emerge from the revelation of misattributed paternity. NG15 recalled three instances of misattributed paternity in a refugee resettlement center. These results not only ended the application process for the fathers in the cases but they often brought up trauma from rape among the women in the population served by the center.

The sensitive information that can be revealed by DNA testing includes more than misattributed parentage. In transnational missing persons cases, while finding a genetic relationship between an FRS and a UHR sample is a step toward identification, it also potentially confirms the death of a relative. The sensitivity of this information was particularly clear in case 27, where a family did not accept the DNA identification of a deceased migrant as their missing child. DNA identification is not unquestionable, nor is a relationship test. In fact, families should be permitted to question results of the tests. In case 27, the family’s rejection of the identification could have stemmed from both emotion and their knowledge of their family member’s case, or their reaction could reflect the sensitivity of communications in this context.

Not every case involving misattributed parentage results in a denial, but the revelation of sensitive information can still be traumatic even in an ultimately successful case. In case 12, a father with an established caregiving relationship with his child came forward as a sponsor when the child was held as an unaccompanied minor in Office of Refugee Resettlement (ORR) custody. A DNA test requested during the application process revealed that he was not the genetic father. This case example also captures the lack of preparedness to communicate unexpected results, as narrated by NG06:

In that case, the government also agreed to leave the decision to disclose with the putative parent, or the caretaker parent, so that he could decide when and how to tell his daughter that there was no biological relationship, because I think they [the ORR] had considered telling her while she was detained and in custody without anyone to be around her to support her to, through learning that information. As if detention isn’t difficult enough for a kid.

Ultimately, the existing caregiving relationship prompted ORR to release the child to him. The couple in case 22, described above, never intended to learn which child was related to which father. The decision by the State Department to grant citizenship to only one child forced them to reveal which child was genetically related to whom, information they had planned to never share, to the government and ultimately the public.

#### Pitfall C: Collection, processing, and comparison of DNA samples from multiple individuals can carry logistical, temporal, geographical, and financial burdens

In some of the cases described, DNA testing was burdensome not because of any inherent characteristic of the process but because of the circumstances in which families had to carry out testing. For instance, while IA13 described the current costs of DNA testing as “reasonable,” five other participants (NG02, IA10, IA11, IA12, and NG15) all contextualized the financial burden of DNA testing within the means of families, particularly low-income and refugee families. They also described instances where DNA testing created burdens due to the structure and history of a family. IA12 noted that the financial burden of testing might be dependent on how many relationships a family needed to test. In case 14, IA23 hoped to compare a client’s DNA data to several half-siblings, but a test had yet to be completed because the siblings were dispersed. Similarly, the adoptee in case 23 spent over a year tracking down his biological siblings in the hopes of using a DNA test as evidence of his US citizenship. Location of family members can also exacerbate challenges that make DNA testing burdensome. In case 13, a young child was left without a proper guardian upon the death of the mother; sample collection in this circumstance was challenging, as the father was in the United States and the child was in a refugee camp abroad. Failure of governments or other organizations to provide appropriate resources or infrastructure also creates burdens. In case 24, border closures and travel restrictions due to the coronavirus prevented a couple from promptly gathering the necessary evidence to finalize an adoption. In addition, the US Embassy in the country where one birth parent was located was reticent to facilitate DNA testing. In the transnational missing context, families might face open-ended waiting times for kinship associations even after samples have been collected and submitted. In case 27, UHR were first exhumed and sampled 7 years after burial; an association was first made about a year after DNA data from the UHR sample were uploaded to a database. In case 28, a DNA sample was collected by a medical examiner and genotyped for STRs, but no kinship matches have yet resulted.

#### Pitfall D: DNA from the appropriate individuals to test a relationship is not always available

This might be due to a death, as in case 14, or to other challenges to sample collection and comparison. IA23 cited a request for a specific antigen test of a decedent as “…an example of…how little prepared the court, to the—the USCIS officers are really. How little informed they are of what some of these tests mean.” In the transnational missing context, FRSs and UHR samples must be entered into the same database for an association to be made. In cases 28 and 29, DNA samples were collected from UHR, but no kinship matches had resulted, potentially because the families had not provided samples, or they had provided samples to a database that was not compared to the one holding the DNA data from the remains.

#### Pitfall E: The appropriate technology and/or infrastructure to test a relationship is not always available

This might include stakeholders’ understanding of testing methodologies, established procedures for requesting and submitting DNA test results, systems for locating and collecting samples, or data management challenges. IA23 described the reaction of USCIS in case 14: “So, we did the blood test with my client who was father/mother A with a half sibling father/mother B and another half sibling father/mother C to establish that, you know, he was, you know, was the father. And USCIS couldn’t wrap its head around that.” IA23 expressed surprise that “they had difficulty” with complex kinship methodologies for demonstrating paternity. NG06 recalled a series of difficult cases out of one East African country, captured in case 15. This country had no paperwork available for ordering a DNA test and no paperwork or system for locating relatives. In case 16, DNA testing was successfully carried out, but submission to USCIS posed a problem. The client and his attorney (IA17) submitted a DNA test before it was requested by USCIS, knowing they had no other documentation of a father-child relationship, but USCIS responded with a request for a birth certificate listing the names of both parents. There did not seem to be a structure in place to allow DNA test results to be submitted up front. TC08 and 09 noted, “Generally the hearsay that I have would be that this [process is] document-driven and then DNA is [requested] towards the end. Documents failed and…then they’ll go to the DNA.” For transnational missing persons, as in unsolved cases 28 and 29, even if FRSs and UHR samples were genotyped and databased, current database structures and policies created information silos that decreased the likelihood of successful associations being made.

#### Pitfall F: The government might not collect samples or request or apply DNA testing results uniformly in decision-making processes

Specific cases described by participants capture how inconsistencies in DNA testing and decision-making processes might manifest. Inconsistencies included instances where DNA tests were requested to evaluate explicitly non-genetic relationships, where the study team found contrasts between the use of DNA in similar cases or where participants felt DNA evidence was weighted in an unexpected or precedent-breaking way, and where policies or laws around the collection, processing, or evaluation of DNA evidence were not followed. Case 19 provides an example of an unreasonable request for DNA evidence, in which USCIS erroneously requested a DNA test of a husband-wife couple. Their attorney (NG21) brought the request to the attention of a USCIS officer, who attributed the request to clerical error. A series of cases where DNA evidence was used to determine with whom a child could reside demonstrates the shifting value placed on paper documentation, histories of caregiving relationships, and DNA evidence. In case 21, NG02 suggested that new DNA requirements put in place by HHS complicated the reunification of parents and children separated under the zero-tolerance immigration policy. While HHS required DNA testing for government-separated children in case 21, in case 12 in the case of an unaccompanied youth, a caregiving relationship was sufficient to release a child to a sponsor despite negative paternity results. In the visa petition context, in both cases 16 and 17, fathers with little or no contact with their children submitted petitions but had little access to paper documentation because they had fled wars. In case 16, results of paternity submitted ahead of a request were not considered sufficient evidence, and in case 17, NG15 was concerned that DNA testing would not help the petition since the father and child had long been estranged and there was little paper documentation available. IA13 highlighted a scenario (case 18) in which, despite DNA evidence of paternity, a father’s petition for his child was rejected based on the law in the country of origin, which required demonstrated financial support and cohabitation. The decision was ultimately overturned in accordance with US law. In contrast, in case 23 a man remained in danger of being deported despite submitting DNA evidence of his biological relationship with six full siblings, all born to two US citizen parents, together with medical records, a birth certificate, documents related to his name change upon adoption, and statements from his adoptive and biological family members. Some case descriptions indicated that government or state officials themselves obstructed DNA collection. In case 24, described above, a US Embassy did not cooperate in the DNA collection required to finalize an intercountry adoption. In addition, in the transnational missing persons context, in cases 27, 29, 30, and 31, DNA samples were initially not collected from UHR despite state law requiring DNA sampling of unidentified corpses.

Participant reflections on the weight of DNA testing as evidence captured additional inconsistencies. Participants relayed contrasting assessments of how they might advise clients and how they thought the government weighted DNA evidence. These assessments were closely interwoven with the perceived reliability of evidence, particularly in connection with characteristics of families and their countries of origin. IA11 stated,

It’s [DNA evidence] heavily, heavily weighted. The government has never ever pushed back on a DNA test result to me. …They’ve pushed back on all kinds of other documents we’ve submitted, but not the DNA test, they have never pushed back about its credibility. …And then I’ve—I’ve always found it interesting; how do they know this company is reputable? Right.

IA12 stated that they always advised DNA testing if financially possible when paper documentation dating a child’s birth was unavailable; in the absence of strong paper documentation, USCIS sees DNA as the “most conclusive evidence.” IA19 reflected a more even weight of DNA evidence in comparison to other forms of documentation, indicating that “it weighs the same” and that “a birth certificate with the father’s name on it that is, you know, authenticated and it’s real is, is acceptable evidence. Again, that’s not—you know, just like a DNA test, that’s not 100 percent either, you know”? Similarly, IA01 described DNA testing as “one tool in an entire toolbox of ways in which to make a connection.”

Differences in the weighting of DNA testing take on significance when consent and discrimination are considered. AC03 noted that while there are contexts where DNA testing was

a reasonable and appropriate…measure…. Where we potentially get into trouble is in situations where the agency begins demanding DNA testing to verify family relationships, not because it thinks there might be a reason for doubt on [a] case-by-case basis, but just because it’s decided than an entire class of cases, like the case—you know, refugees from South West Africa, say—[might be fraudulent].

The concept of “high-fraud countries” (IA04) ran throughout several interviews to varying degrees. IA11 drew a relationship between requests for DNA testing, other forms of documentation, the reputation of certain regions, and the possibility of discrimination. He related that “in the vast majority of cases that I’ve used DNA testing to prove biological relationships, they were—the people were of African descent…um, and Black African descent, not white South Africa or North African Arabs. They were Black Africans.” When asked why that might be, IA11 responded, “There’s a tremendous amount of doubt about the paperwork that comes from a lot of those countries in Africa. The general or common thought is that…there’s a lot of fraudulent documentation being purchased and used.” He concluded, “And so, those countries that are less developed, the record-keeping is less trusted, then the government will push back and you have to use DNA testing to prove the relationships.” IA10 raised the issue of consent for the families that are presented with requests for DNA testing:

I don’t feel—I feel like if immigration requests it, I don’t feel like you have an option to say no. Because if you say no, then they’re going to infer that it’s not a real relationship…. And so I don’t think there are, you know, circumstances where I would tell a client not to do it. Because they’ve already assured me that this person is their child and everything, so you know, to some degree if they want the case approved, I mean they have to get—they have to do it. You know? And I am not sure that there’s really a choice.”

## Discussion

Our findings provide insight into the distinct utility of genetic information in a non-medical context, as well as the potential pitfalls of using genetic information as a proxy for family relationships. They also illustrate the interplay of utilities and pitfalls in individual cases and how they affect petitioners and professional stakeholders. By identifying utilities and pitfalls in known cases and pinpointing those that could apply across contexts, we provide a framework to aid in the development of guidelines in emergent application contexts.

We were unable to access the full range of stakeholders who might be involved with DNA testing for immigration, particularly government officials or agents, for interviews. The challenges of identifying and contacting government officials aligns with the lack of transparency about the details of DNA testing processes in immigration contexts. Our information-oriented sampling yielded an insufficient number of immigration attorneys, which prompted our adjusted sampling approach. Most cold email invitations went unanswered, but some responded that they did not have experience with DNA testing, despite our instruction that experience was not a requisite for participation. This speaks to the lack of broad uptake of DNA tests in immigration practices and possibly to the ignorance of the availability of DNA tests as a source of evidence, although not all immigration law involves family petitions. The immigration attorneys also expressed the limits of their knowledge and experience of DNA testing despite working in the longest-standing DNA testing context. The combination of their emphasis of their lack of experience and assertions of the power of DNA evidence when used also indicates a need for tools for understanding the potential implications of the technology.

Our interviews also did not capture case examples for every context. Tapping into news sources enabled us to highlight the utilities and pitfalls relevant to some of those contexts, but a systematic media analysis or further interviews would likely expand the understanding of these contexts. Nevertheless, our framework is rooted in qualitative analysis of the experiences of the participants. Further interviews are also warranted by the ongoing rapid developments in this sphere.

The potential utility of a DNA test in helping a family support their immigration case, particularly when initiated by the family (rather than required by authorities), might well balance the risk of some of the pitfalls arising, depending on the case. Inflexible definitions of family and lack of transparency around DNA testing applications in immigration, however, remain primary concerns. A broader definition of family^5^ is needed in immigration policies to avoid some of the pitfalls outlined in our results. DNA testing should be used in tandem with, not in place of, other forms of documentary evidence and should not by itself be used to support exclusion from immigration benefits. A lack of transparency in how DNA tests are weighted in cases and what is termed fraud across immigration contexts hampers the development of guidelines that draw on input from the broad array of implicated stakeholders. Lack of transparency on how the government defines fraud in terms of family units compounds the pitfalls of applying DNA data to these cases.^8^ In the rapid DNA testing for family relationship verification at border entry points, it remains unclear how families are selected for testing and evaluated for fraud.^27^

As Holland^5^ asserts, inflexible definitions of family are particularly prone to disadvantage migrant families, who endure circumstances that disrupt and re-form relationships of all kinds. With greater understanding of the processes, migrant advocates can better guide families on when DNA tests are necessary, and inconsistencies in the weight of DNA test results on decisions might dissipate. The balance of benefits and risks here is akin to the balance considered in health-related uses of genetic information to guide consent and pre- and post-communication of results. Both health-related and long-standing immigration contexts can provide a wealth of understanding to inform guidelines that allow access to the utility of relationship DNA testing in immigration contexts while minimizing pitfalls.

## Data and code availability

There are restrictions to the availability of the interview data due to participant confidentiality and risks to the people whose immigration cases and family dynamics were discussed. Data are protected with a Certificate of Confidentiality. Case example narratives and citations to sources for cases in the public domain are available in supplemental information. Authors may contact the study team for de-identified transcripts.

## Web resources

DNA Bridge, https://www.dnabridge.org/
Rev, https://www.rev.com
American Immigration Lawyers Association, https://www.aila.org

## Supplemental information

Document S1. Supplemental materials and methods and supplemental notes

Document S2. Article plus supplemental information
