# Supplementary material for: Framing the utility and potential pitfalls of relationship and identity DNA testing across United States immigration contexts
Source: HGG Adv. 2021 Sep 24;3(1):100060. doi: 10.1016/j.xhgg.2021.100060 (PMC8756547; doi:10.1016/j.xhgg.2021.100060)
Supplement: Document S1. Supplemental materials and methods and supplemental notes [file mmc1.pdf]

HGGA, Volume *3*

Supplemental information

Framing the utility and potential pitfalls of relationship and identity DNA testing across United States immigration contexts

Diana Madden, Brianna A. Baker, Jennifer K. Wagner, and Sara H. Katsanis

Exhibit EI-G - Expert interview guide (*April 2018 protocol*)

INTERVIEWER: ________________________ STUDY ID: ________________________

DATE: ______________________

Thank you for agreeing to talk with us. As stated in the [email / letter / phone call] you received from us, we are collecting feedback from expert stakeholders who work with migrant families that may be providing DNA samples or undergo DNA tests. Our research team is conducting interviews with a number of people and we are interested in your opinions and experiences.

[REVIEW CONSENT FORM AS PER PROTOCOL]

1. To get things started, can you tell us what you know about how DNA tests are used in immigration?

2. What is your involvement with migrants or migrant families?

3. Do you order or help arrange DNA [tests / sample collection] for migrants or families?

4. [IF Q2 YES] What kind of DNA tests do you order? Who performs the tests? Who pays for tests?

5. Briefly, what is your understanding of what a DNA test will tell you?

6. How do you expect DNA test results to be helpful?

7. In what circumstances do you not suggest DNA tests?

8. Do you receive DNA test results?

9. [IF Q7 YES] With whom might you share the DNA test results?

10. Do you think there are any risks in taking a DNA test?

11. Likert Scale: On a scale of 1 to 5, how important DNA testing in immigration? With “1” being not important at all and “5” being very important

That concludes our session. Thank you so much for sharing your thoughts and opinions with us. If you have additional information that you did not get to say today, please contact one of us.

CASE EXAMPLE CATALOG

PARTICIPANT-SOURCED CASE EXAMPLES **2**
CONTEXT 1: DNA TESTING FOR RELATIONSHIP OR IDENTITY VERIFICATION FOR VISA OR CITIZENSHIP APPLICATIONS AND PETITIONS FOR NONCITIZEN RELATIVES 3
Case 1: DNA testing documents a relationship *3*
Case 2: DNA testing documents a relationship *3*
Case 3: DNA testing documents a relationship *4*
Case 4: DNA testing disproves fraud *4*
Case 5: DNA testing detects fraud *5*
Case 6: DNA testing detects fraud *5*
Case 7: DNA testing requests deter fraud *5*
Case 8: DNA testing disproves fraud and reveals sensitive information *6*
Case 9: DNA testing reveals misattributed paternity *6*
Case 10: DNA testing reveals misattributed paternity *7*
Case 11: DNA testing reveals misattributed paternity *7*
Case 12: DNA testing reveals misattributed paternity *8*
Case 13: DNA testing is burdensome, but documents a relationship *8*
Case 14: DNA testing is burdensome, but could document a relationship *9*
Case 15: DNA testing is burdensome *9*
Case 16: DNA documentation of a relationship is insufficient *10*
Case 17: DNA documentation of a relationship could be insufficient *11*
Case 18: DNA documentation of a relationship is insufficient *11*
Case 19: DNA testing is requested erroneously *12*
CONTEXT 2: DNA TESTING FOR RELATIONSHIP VERIFICATION FOR PLACEMENT WITH SPONSORS OF UNACCOMPANIED MIGRANT MINORS 13
Case 20: DNA testing is not requested uniformly across similar cases *13*
CONTEXT 3: DNA TESTING FOR VERIFICATION OF PARENT-CHILD RELATIONSHIPS FOLLOWING GOVERNMENT-IMPOSED FAMILY SEPARATION 14
Case 21: DNA testing is burdensome *14*
STUDY TEAM-SOURCED CASE NARRATIVE EXAMPLES **15**
CONTEXT 1: DNA TESTING FOR RELATIONSHIP OR IDENTITY VERIFICATION FOR VISA OR CITIZENSHIP APPLICATIONS AND PETITIONS FOR NONCITIZEN RELATIVES 16
Case 22: DNA testing is requested erroneously *16*
Case 23: DNA documentation of a relationship is insufficient *17*
Case 24: Officials will not collect a DNA sample *18*
CONTEXT 4: DNA TESTING FOR VERIFICATION OF PARENT-CHILD RELATIONSHIPS AT BORDER ENTRY POINTS 19
Case 25: DNA testing request reveals fraud *19*
CONTEXT 5: COMPARISON OF FAMILY REFERENCE SAMPLES TO UNIDENTIFIED HUMAN REMAINS SAMPLES FOR IDENTIFICATION PURPOSES IN TRANSNATIONAL MISSING PERSONS CASES 20
Case 26: DNA testing documents a genetic relationship *20*
Case 27: DNA testing documents a relationship, but is rejected by the family *20*
Case 28: Infrastructure delays or prevents a DNA identification *21*
Case 29: Infrastructure delays or prevents a DNA identification *22*
Case 30: Infrastructure delays or prevents a DNA identification *23*
Case 31: Officials will not collect a DNA sample *23*

This report was prepared as part of the Genomics, Biometrics, and Identity project, at Lurie Children's Hospital and Northwestern University. Team members include SH Katsanis, D Madden, JK Wagner, D Berger, MK Spradley, E Canales, B Baker, A Porter, and Z Guzman. This project was funded by the National Human Genome Research Institute R01HG009923.

PARTICIPANT-SOURCED CASE EXAMPLES

The following case example narratives highlight the utilities and pitfalls of DNA testing for relationships and identity in three of five U.S. immigration contexts where it is applied. These narratives were developed from descriptions of specific cases in interviews with professional stakeholders in immigration in the United States conducted in 2019. Each case narrative has been coded with the applicable overarching utilities and pitfalls of DNA testing. Codes were assigned from the perspective of the participant, based on participant statements about the specific utility and pitfalls of DNA testing in the case.

All case examples have been anonymized. Names are not included and are replaced with relevant kinship terms or a randomly assigned letter where necessary. Dates and gender are only included where judged necessary to support the narrative. Where a country of origin outside of the United States was specified, we include the region of the world where the country is located. All case examples occurred in the context of the U.S. immigration system.

Participant-sourced case examples are presented in the following format:

Assigned case number: Descriptive caption

**Source**	Code assigned to participant (participant's profession and assigned number)
**Region**	Region of the world where country or countries involved in the case are located (excluding the United States)
**DNA test type**	Retrospective/prospective DNA test and laboratory type OR DNA test and laboratory type that would have been used had testing been carried out
**Relationship tested**	Familial relationship(s) (to be) tested
**Stated value**	Utility of DNA testing in the case based on participant statements
**Stated pitfall(s)**	Pitfall(s) of DNA testing in the case based on participant statements
**Narrative**	Anonymized narrative of the case developed from interview transcripts and follow-up with participants where required

CONTEXT 1: DNA testing for relationship or identity verification for visa or citizenship applications and petitions for noncitizen relatives

Case 1: DNA testing documents a relationship

**Source**	Interview with Immigration Attorney 19 [IA19]
**Region**	Unspecified
**DNA test type**	Relationship STR at a commercial AABB laboratory
**Relationship tested**	Father-child
**Stated value**	DNA testing can provide documentation of genetic relationships and identity when other forms of documentation are unavailable, inaccurate, or insufficient to meet the burden of evidence.
**Stated pitfall(s)**	No pitfalls stated
**Narrative**	A U.S. citizen child wished to petition for a non-U.S. citizen father to obtain a green card for the parent. The father was not listed on the child's birth certificate, however, so a DNA test was used to establish the biological relationship.

Case 2: DNA testing documents a relationship

**Source**	Interview with Immigration Attorney 04 [IA04]
**Region**	North America
**DNA test type**	Relationship STR at a commercial AABB laboratory
**Relationship tested**	Mother-child
**Stated value**	DNA testing can provide documentation of genetic relationships and identity when other forms of documentation are unavailable, inaccurate, or insufficient to meet the burden of evidence.
**Stated pitfall(s)**	No pitfalls stated
**Narrative**	A woman, hereafter referred to as A, had a child out of wedlock in her home country while still a teenager. Because A was unmarried, her parents insisted that they be listed as the parents on their grandchild's birth certificate. Both A and her child had the exact same family name: on paper, they appeared to be full siblings with the same family name and a large age gap between them. Both A and her child became eligible for a special visa for victims of crime, which could ultimately lead to a green card. Because A's child was still a minor, her attorney filed on behalf of A; however, for both A and her child to get the visa, it had to be established that they had a mother-child (not sibling) relationship. DNA testing confirmed that their relationship was mother and child.

Case 3: DNA testing documents a relationship

**Source**	Interview with NGO Representative 21 [NG21]
**Region**	North Africa and Central Africa
**DNA test type**	Relationship STR at a commercial AABB laboratory
**Relationship tested**	Mother-child
**Stated value**	DNA testing can provide documentation of genetic relationships and identity when other forms of documentation are unavailable, inaccurate, or insufficient to meet the burden of evidence.
**Stated pitfall(s)**	No pitfalls stated
**Narrative**	A husband and wife were processed for refugee status in the United States while in a North African country; the husband was named the principal applicant. Their marriage was found valid, and they entered the United States together. Once in the United States, they decided to petition for the wife's biological children (the husband's stepchildren), whom they had left behind in a third country in Central Africa, to join them. The husband, having been named the principal applicant for refugee status, had to be the one to file the petition even though the children were not biologically his. The wife had to consent to a DNA test to show that the children were biologically hers; because the marriage had already been deemed valid, the DNA test confirmed that the petitioner was the stepfather.

Case 4: DNA testing disproves fraud

**Source**	Interview with Immigration Attorney 11 [IA11]
**Region**	West Africa
**DNA test type**	Relationship STR at a commercial AABB laboratory
**Relationship tested**	Unclear
**Stated value**	DNA testing can disprove or detect fraud when claimed relationships or identity are in question.
**Stated pitfall(s)**	No pitfalls stated
**Narrative**	An attorney had a U.S. citizen client who had gone through the process of naturalization in the United States. U.S. Citizenship and Immigration Services [USCIS] claimed that the client was not who they said they were and had not been eligible for the lawful permanent residence or green card status that gave rise to naturalization. The government thus wanted to denaturalize the person and rescind their lawful permanent resident status. The attorney had the client use a DNA test to verify their identity, thereby demonstrating that they were eligible for the status that originally led to naturalization.

Case 5: DNA testing detects fraud

**Source**	Interview with Technology Company Representatives 07 and 08 [TC08 and TC09]
**Region**	South Asia
**DNA test type**	Relationship STR at a commercial AABB laboratory
**Relationship tested**	Brother-sister
**Stated value**	DNA testing can disprove or detect fraud when claimed relationships or identity are in question.
**Stated pitfall(s)**	No pitfalls stated
**Narrative**	The DNA of a man and woman claiming to be brother and sister was tested, and the profiles derived from the samples were both male. Since the second sample should have been female, the laboratory requested a resampling, and the results profiled the same way. It was suspected that somebody was substituting the samples to cover up the unrelatedness of the two supposed siblings.

Case 6: DNA testing detects fraud

**Source**	Interview with Technology Company Representative 07 [TC07]
**Region**	Unspecified
**DNA test type**	Relationship STR at a commercial AABB laboratory
**Relationship tested**	Parent-child
**Stated value**	DNA testing can disprove or detect fraud when claimed relationships or identity are in question.
**Stated pitfall(s)**	No pitfalls stated
**Narrative**	A third party in an unspecified country was submitting the same known related samples to different laboratories for multiple cases. The State Department reviewed the results from several different individuals from that same country and realized that the same profile had been used repeatedly.

Case 7: DNA testing requests deter fraud

**Source**	Interview with Immigration Attorney 18 [IA18]
**Region**	Unspecified
**DNA test type**	Relationship STR at a commercial AABB laboratory
**Relationship tested**	Avuncular
**Stated value**	DNA testing requests or requirements can deter intentionally fraudulent misrepresentations of relationships or clarify misunderstandings around the kinds of relationships that qualify for immigration benefits.

**Stated pitfall(s)**	Family is not defined by genetic relationships alone; kinship terms do not correspond to biological (or genetic) relationships in the same way across languages and cultures.
**Narrative**	An attorney has had cases where clients revealed after receiving a government request for a DNA test that they were petitioning for biological nieces or nephews, not biological children. The attorney stated that sometimes it seemed to be intentional concealment of the true relationship by the petitioner and sometimes misunderstanding of the documentation (and relationship) needed to support their petitions. The attorney recommends to clients who are not the biological parents of a child to withdraw their applications rather than comply with requests for DNA testing that could reveal non-parentage and thereby risk being accused of fraud.

Case 8: DNA testing disproves fraud and reveals sensitive information

**Source**	Interview with Immigration Attorney 01 [IA01]
**Region**	South Asia
**DNA test type**	Relationship STR at a commercial AABB laboratory
**Relationship tested**	Mother-child
**Stated value**	DNA testing can disprove or detect fraud when claimed relationships or identity are in question.
**Stated pitfall(s)**	Kinship analysis can reveal sensitive information.
**Narrative**	In South Asia, the husband of a woman died while their baby was an infant. The husband's family took the child and told the woman that the baby also had died. Not realizing her child was still alive, the woman eventually immigrated to the United States, where she remarried. Decades later, she was contacted by an individual from South Asia who thought they were her child; the individual said they had been looking for her for the past 15 years. Because of the unusual circumstances, her attorney recommended a DNA test to make sure the claim was not a scam. The person turned out to be her biological child, and she filed a petition on their behalf. The question emerged as to whether the woman had committed fraud in the past by not disclosing the existence of the child, whom she had presumed dead.

Case 9: DNA testing reveals misattributed paternity

**Source**	Interview with Immigration Attorney 01 [IA01]
**Region**	West Africa
**DNA test type**	Relationship STR at a commercial AABB laboratory
**Relationship tested**	Father-child
**Stated value**	No utility stated
**Stated pitfall(s)**	Kinship analysis can reveal sensitive information.

**Narrative**	A married man living in the United States believed he had fathered child abroad out of wedlock. He revealed the existence of the child to his wife and decided he wanted to bring the child to the United States. He opened a petition, and a DNA test was requested. The results unexpectedly revealed that the child was not his biological child. Because he had no legal connection to the child (he was not married to the child's mother) and ultimately no biological connection, the petition was denied. His immigration attorney suggested that he could go through a separate process to adopt the child to establish a legal relationship.

Case 10: DNA testing reveals misattributed paternity

**Source**	Interview with NGO Representative 21 [NG21]
**Region**	Central America
**DNA test type**	Relationship STR at a commercial AABB laboratory
**Relationship tested**	Father-child
**Stated value**	No utility stated
**Stated pitfall(s)**	Kinship analysis can reveal sensitive information.
**Narrative**	A father applied to bring his child to the United States through the Central American Migrant Minors [CAM] program. The mother was in the country of origin, and the father was in the United States. The father, realizing he had the opportunity to bring his child to a safe place through the CAM program, petitioned for the child. DNA testing was required and revealed he was not the biological father; he thus had no recourse to get the child to the United States. His immigration attorney reportedly was not prepared to give him results of non-paternity.

Case 11: DNA testing reveals misattributed paternity

**Source**	Interview with NGO Representative 15 [NG15]
**Region**	Unspecified
**DNA test type**	Relationship STR at a commercial AABB laboratory
**Relationship tested**	Father-child
**Stated value**	No utility stated
**Stated pitfall(s)**	Kinship analysis can reveal sensitive information.
**Narrative**	A coordinator in a refugee resettlement center recalled at least three instances of misattributed paternity, which in the population they worked with was generally connected with rape. They described three cases in which fathers believed that children were biologically theirs, but DNA testing revealed misattributed paternity. Reporting misattributed paternity would often cause women to revisit rape trauma in the population the center served. In these cases, the

	coordinators would sit down and talk about the DNA test results with the clients. Misattributed paternity put an end to the immigration application process for the fathers.

Case 12: DNA testing reveals misattributed paternity

**Source**	Interview with NGO Representative 06 [NG06]
**Region**	Unspecified
**DNA test type**	Relationship STR at a commercial AABB laboratory
**Relationship tested**	Father-child
**Stated value**	No utility stated
**Stated pitfall(s)**	Kinship analysis can reveal sensitive information. The government might not request or apply DNA testing results uniformly in decision-making processes.
**Narrative**	While a DNA test that verifies a claimed biological relationship between a child and someone stepping forward to claim that child from ORR weighs heavily with the government, one participant described a case where a care-giving relationship was sufficient to convince ORR to release a child to a sponsor. A father and child had a 15-year relationship and believed themselves to be biologically related, although the father was not entirely certain he was the biological father. DNA testing ultimately showed that they were not genetically related, but the fact that they had a care-giving father-child relationship, if not a biological one, and that he was the safest and most appropriate placement for the child convinced the government to release the child to him. In this case, the government left the decision to disclose the results of the DNA test to the child up to the father, although the government also initially considered informing the child while they were in detention.

Case 13: DNA testing is burdensome, but documents a relationship

**Source**	Interview with Immigration Attorney 17 [IA17]
**Region**	East Africa
**DNA test type**	Relationship STR at a commercial AABB laboratory
**Relationship tested**	Father-child
**Stated value**	DNA testing can provide documentation of genetic relationships and identity when other forms of documentation are unavailable, inaccurate, or insufficient to meet the burden of evidence.
**Stated pitfall(s)**	Collection, processing, and comparison of DNA samples from multiple individuals can carry logistical, temporal, geographical, and financial burdens.
**Narrative**	A man came to the United States and petitioned for his wife and child to join him. The wife and child were in a refugee camp abroad; the

	wife died of malaria in the camp, leaving the child in the care of a very young relative and without a proper guardian. The birth certificate issued by the refugee camp was not considered sufficient evidence of the relationship between the father and child by USCIS; USCIS requested a birth certificate from the country the family fled as well as DNA evidence. Coordinating the logistics of obtaining DNA from the child in the refugee camp was extremely difficult given the circumstances. The petition was ultimately approved when DNA testing verified the father's relationship with the child.

Case 14: DNA testing is burdensome, but could document a relationship

**Source**	Interview with Immigration Attorney 23 [IA23]
**Region**	North America
**DNA test type**	Relationship STR at a commercial AABB laboratory
**Relationship tested**	Father-child; half siblings
**Stated value**	DNA testing can provide documentation of genetic relationships and identity when other forms of documentation are unavailable, inaccurate, or insufficient to meet the burden of evidence.
**Stated pitfall(s)**	Collection, processing, and comparison of DNA samples from multiple individuals can carry logistical, temporal, geographical, and financial burdens. DNA from the appropriate individuals to test a relationship is not always available. The appropriate technology and/or infrastructure to test a relationship is not always available.
**Narrative**	A client claiming to be a U.S. citizen had a U.S. citizen father who had died, leaving behind the client and several alleged full and/or half-siblings. The client was deported several times by U.S. authorities despite having a birth certificate with the alleged father named. The identity of the client's mother was disputed as one of two sisters, but the alleged mothers also were deceased. Since the client's parents were all deceased, the attorney suggested a DNA test of the half-siblings related through the father along with the client to demonstrate the paternal relationship. However, since the client had been repeatedly deported and the siblings were dispersed, coordinating the DNA testing of the various parties was difficult to accomplish, and testing had yet to be completed. It was unclear whether DNA evidence from multiple siblings would be successful in demonstrating the client's paternal relationship and claim to citizenship.

Case 15: DNA testing is burdensome

**Source**	Interview with NGO Representative 06 [NG06]
**Region**	East Africa
**DNA test type**	Relationship STR at a commercial AABB laboratory

**Relationship tested**	Unspecified
**Stated value**	No utility stated
**Stated pitfall(s)**	Collection, processing, and comparison of DNA samples from multiple individuals can carry logistical, temporal, geographical, and financial burdens. The appropriate technology and/or infrastructure to test a relationship is not always available.
**Narrative**	An NGO worker described a series of difficult cases out of a country in East Africa. The worker indicated that there was no paperwork available for processing DNA testing and finding relatives in the country, but even more challenging, there was no system for finding family members to conduct DNA tests. They indicated that very often relationships were established through means other than DNA testing, such as interviews.

Case 16: DNA documentation of a relationship is insufficient

**Source**	Interview with Immigration Attorney 17 [IA17]
**Region**	Unspecified
**DNA test type**	Relationship STR at a commercial AABB laboratory
**Relationship tested**	Father-child
**Stated value**	DNA testing can provide documentation of genetic relationships and identity when other forms of documentation are unavailable, inaccurate, or insufficient to meet the burden of evidence.
**Stated pitfall(s)**	The appropriate technology and/or infrastructure to test a relationship is not always available. The government might not collect samples or request or apply DNA testing results uniformly in decision-making processes.
**Narrative**	A father, now a naturalized U.S. citizen, fled civil war in his home country, leaving behind his child. Because he was forced to flee, he was not present at the birth of the child and was not listed on the child's birth certificate. He had, however, maintained contact with the child. After the child had grown a bit older, the father decided to petition for the child to join him. The father and attorney decided to submit a DNA test upfront, knowing that they lacked other forms of documentation of the relationship and knowing that the father had the means to pay for a DNA test. A DNA test was done, confirmed the claimed relationship, and was submitted to USCIS. USCIS sent a request for further documentation even though they already had the DNA test as proof of the relationship; specifically, they asked for a birth certificate with the names of both parents.

Case 17: DNA documentation of a relationship could be insufficient

**Source**	Interview with NGO Representative 15 [NG15]
**Region**	East Africa
**DNA test type**	Relationship STR at a commercial AABB laboratory
**Relationship tested**	Father-child
**Stated value**	DNA testing can provide documentation of genetic relationships and identity when other forms of documentation are unavailable, inaccurate, or insufficient to meet the burden of evidence.
**Stated pitfall(s)**	The government might not collect samples or request or apply DNA testing results uniformly in decision-making processes.
**Narrative**	Due to war, a father from East Africa living in the United States had been estranged from his child since the child was six months old. The child's mother was dead. Due to the estrangement, there was little documentation outside of a DNA test to establish the claimed relationship. The attorney representing the child questioned whether a DNA test would be relevant or useful in establishing with whom the child could reside, since the child does not know the father.

Case 18: DNA documentation of a relationship is insufficient

**Source**	Interview with Immigration Attorney 13 [IA13]
**Region**	Caribbean
**DNA test type**	Relationship STR at a commercial AABB laboratory
**Relationship tested**	Father-child
**Stated value**	DNA testing can provide documentation of genetic relationships and identity when other forms of documentation are unavailable, inaccurate, or insufficient to meet the burden of evidence.
**Stated pitfall(s)**	The government might not collect samples or request or apply DNA testing results uniformly in decision-making processes.
**Narrative**	A man from a Caribbean country living in the United States petitioned for his child (a minor) to join him; he did all the paperwork himself as he could not afford counsel. He was an American citizen, but his name did not appear on the child's birth certificate. A request for DNA testing was issued, authorized, and properly carried out by USCIS. DNA testing confirmed the father and child's genetic relationship. Despite the DNA match, the petition was denied by the United States based on the law in the country of origin, which required demonstrated financial support of the child and a demonstrated period of cohabitation. Upon appeal, the decision was overturned in favor of uniform application of U.S. law to petitions without regard to legal qualifications for parenthood in the country of origin.

Case 19: DNA testing is requested erroneously

**Source**	Interview with NGO Representative 21 [NG21]
**Region**	East Africa
**DNA test type**	Relationship STR at a commercial AABB laboratory
**Relationship tested**	Husband-wife
**Stated value**	No utility stated
**Stated pitfall(s)**	The government might not collect samples or request or apply DNA testing results uniformly in decision making processes.
**Narrative**	A husband and wife applied to come to the United States and received a request for DNA evidence from a USCIS service center. Their attorney found it odd, but they ultimately complied. When the attorney brought the request to the attention of a USCIS officer, the officer agreed that it was inappropriate and said that they would do retraining to avoid such requests in the future. The attorney initially thought that the request might be due to suspicion of fraud on USCIS's part, especially since the couple's country of origin only began producing civil issue documents accepted by the U.S. government in the mid-2010s; it was conceivable that a brother-sister pair without documentation of their relationship might try to apply as husband and wife. Upon further discussion with a more senior USCIS official, however, the attorney began to suspect that this was not an instance where USCIS suspected fraud, but rather that USCIS personnel had simply copy-pasted evidence requests without regard to the details of the case.

CONTEXT 2: DNA testing for relationship verification for placement with sponsors of unaccompanied migrant minors

Case 20: DNA testing is not requested uniformly across similar cases

**Source**	Interview with Immigration Attorney 16 [IA16]
**Region**	Unspecified
**DNA test type**	Relationship STR at a commercial AABB laboratory
**Relationship tested**	Mother-child
**Stated value**	No utility stated
**Stated pitfall(s)**	The government might not collect samples or request or apply DNA testing results uniformly in decision-making processes.
**Narrative**	An unaccompanied minor presented at the border hoping to go to the mother, located in the United States. The mother was asked to provide DNA to prove the relationship before picking up the child up from the Office of Refugee Resettlement [ORR] housing facility. The attorney stated that in other similar cases, DNA testing was not required for release to a sponsor and was not sure why it was part of the process in this case. The child was held by the U.S. Department of Health and Human Services [HHS] until the relationship was confirmed.

CONTEXT 3: DNA testing for verification of parent-child relationships following government-imposed family separation

Case 21: DNA testing is burdensome

**Source**	Interview with NGO Representative 02 [NG02]
**Region**	Central America
**DNA test type**	Relationship STR at a commercial AABB laboratory
**Relationship tested**	Parent-child
**Stated value**	No utility stated
**Stated pitfall(s)**	Collection, processing, and comparison of DNA samples from multiple individuals can carry logistical, temporal, geographical, and financial burdens.
**Narrative**	After the 2018 separations of children from family members traveling from Central America, one NGO reportedly agreed to take on 63 cases of separated children in attempts to reunify them. ORR used to consider a birth certificate or affirmation enough to release a child from their custody, but HHS had added a DNA requirement that created difficulties for the NGO in reuniting children with parents. Of the 63 family separation cases, only one or two involved relationships other than parent-child. In 15 of the 63 cases, a parent located in the United States wished to claim the child, and DNA testing was required to verify the claimed relationships. The NGO worked to discuss the process with children who were of the age of consent and able to communicate as well as to locate parents in detention. While the majority were reunited with their parents, a handful of them were unable to be reunited and required separate legal remedies to be released, either to a more distant relative, like an aunt or an uncle, or to another parent that was in the United States.

STUDY TEAM-SOURCED CASE NARRATIVE EXAMPLES

The following supplemental case example narratives highlight the utility and pitfalls of DNA testing in three of five U.S. immigration contexts where it is applied. These narratives were developed from cases provided by the study team. Each case narrative has been coded with the applicable overarching utilities and pitfalls of DNA testing. Utilities and pitfalls were interpreted by the study team based on the available information.

Many of these cases are publicly available in academic, media, or government reports; where cases are publicly available, a citation is provided, but the catalogue contents are anonymized as in the participant-sourced case examples. Cases not publicly available are cases in which a member of the study team is directly involved. In all cases, names are not included and are replaced with relevant kinship terms or a randomly assigned letter where necessary. Dates and gender are only included where judged necessary to support the narrative. Where a country of origin outside of the United States was specified, we include the region of the world where the country is located. All case examples occurred in the context of the U.S. immigration system.

Study-team sourced case examples are presented in the following format:

Assigned case number: Descriptive caption

**Source**	Study team reported or communication
**Region**	Region of the world where country or countries involved in the case are located (excluding the United States)
**DNA test type**	Retrospective/prospective DNA test and laboratory type OR DNA test and laboratory type that would have been used had testing been carried out
**Relationship tested**	Familial relationship(s) (to be) tested
**Stated value**	Utility of DNA testing in the case as interpreted by the study team
**Stated pitfall(s)**	Pitfall(s) of DNA testing in the case as interpreted by the study team
**Narrative**	Anonymized narrative of the case developed from details provided by the study team and/or publicly available information. Relevant citations to media, academic, or government reports provided.

CONTEXT 1: DNA testing for relationship or identity verification for visa or citizenship applications and petitions for noncitizen relatives

Case 22: DNA testing is requested erroneously

**Source**	Study team reported
**Region**	North America and Middle East
**DNA test type**	Relationship STR at a commercial AABB laboratory
**Relationship tested**	Father-child
**Interpreted value**	No utility interpreted
**Interpreted pitfall(s)**	Family is not defined by genetic relationships alone; kinship terms do not correspond to biological (or genetic) relationships in the same way across languages and cultures. Kinship analysis can reveal sensitive information. The government might not collect samples or request or apply DNA testing results uniformly in decision-making processes.
**Narrative**[1]	Twins Child 1 and Child 2 were born abroad to a married couple, Father 1 and Father 2, via surrogate using an anonymous donor's eggs and sperm from each father. Father 1 is a U.S. citizen, and Father 2 is a citizen of a Middle Eastern country. When the couple applied for U.S. passports for their newborn children in preparation to return to the U.S., DNA testing was requested to demonstrate both children's connection to Father 1 even though the couple had already been asked to explain how the twins were conceived and were legally married. The couple had planned never to reveal which child was genetically related to whom, even to the children themselves, but carried out the DNA testing hoping to obtain passports for their children. The results demonstrated that Child 1 was genetically related to Father 1 (the U.S. citizen) and Child 2 to Father 2; the U.S. Department of State only granted citizenship to Child 1. Under birthright citizenship laws, children born abroad to one American parent should qualify as citizens, so both children should have been granted citizenship. regardless of the DNA test results. The couple pursued legal action in the U.S. and after several years, a federal court ruled that Child 2 should have been granted citizenship at birth because the parents are legally married. This ruling was in contrast to the Assisted Reproductive Technology and Surrogacy Abroad policy by the

[1] M Flynn. One twin was a citizen, the other undocumented. A victory in court for their same-sex parents rebukes the State Department. *The Washington Post* (22 February 2019). Available at https://www.washingtonpost.com/nation/2019/02/22/one-twin-was-citizen-other-undocumented-victory-court-their-same-sex-parents-rebukes-state-dept/

AD Craythorne. Same-sex equality in immigration law: The case for birthright citizenship for foreign-born children of U.S. citizens in same-sex binational unions. *Texas Law Review* **97**, 645-671 (2019)

A Tchekmedyian. These twins were born 4 minutes apart. But only one is a U.S. citizen. *Los Angeles Times* (27 January 2018). Available at https://www.latimes.com/local/lanow/la-me-ln-twins-citizenship-20180127-story.html

	Department of State that specifies "a U.S. citizen father must be the genetic parent of the child and meet all other statutory requirements in order to transmit U.S. citizenship to the child at birth."[2]

Case 23: DNA documentation of a relationship is insufficient

**Source**	Study team reported
**Region**	North America
**DNA test type**	Relationship STR at a commercial AABB laboratory
**Relationship tested**	Siblings
**Interpreted value**	DNA testing can disprove or detect fraud when claimed relationships or identity are in question.
**Interpreted pitfall(s)**	Collection, processing, and comparison of DNA samples from multiple individuals can carry logistical, temporal, geographical, and financial burdens. The government might not collect samples or request or apply DNA testing results uniformly in decision-making processes.
**Narrative**[3]	A man born in the United States to two U.S. citizens was adopted by a family abroad. After the adoption, the last name on his birth certificate, which was originally both of his biological parents' last names, was changed to reflect both of his adoptive parents' last names. He grew up in the U.S. believing he was born abroad. He was once almost deported but won his case to remain. When immigration officials later attempted to deport him a second time, his adoptive mother told his wife that he was born in the U.S. and instructed her to help him. Fifteen (15) years prior, his adoptive family had found his biological brother's birth certificate; after a year and a half of searching, his adoptive family located not only his biological brother, but also five biological sisters. After USCIS gave him 30 days to prove his U.S. citizenship, his immigration attorney presented "DNA proof of the man's six biological siblings, medical records that prove he was born in a U.S. hospital to two American parents, a foreign birth certificate that indicates he was adopted, documents that show his original last name was changed and statements from his family, including a testament from his biological mother." Due to the changes in his last name, however, he is still in danger of being deported.

[2] U.S. Department of State. Assisted Reproductive Technology (ART) and surrogacy abroad. *U.S. Citizenship Laws and Policy* (2020). Available at https://travel.state.gov/content/travel/en/legal/travel-legal-considerations/us-citizenship/Assisted-Reproductive-Technology-ART-Surrogacy-Abroad.html

[3] M González. Despite being born in the US, adopted man says he is in danger of being deported to Mexico again. *NBC San Diego* (9 February 2021). Available at https://www.nbcsandiego.com/news/local/despite-being-born-in-the-us-adopted-man-says-he-is-in-danger-of-being-deported-to-mexico-again/2516803/

Case 24: Officials will not collect a DNA sample

**Source**	Study team reported
**Region**	West Africa
**Dna test type**	Relationship STR at a commercial AABB laboratory
**Relationship tested**	Parent-child
**Stated value**	DNA testing can provide documentation of genetic relationships and identity when other forms of documentation are unavailable, inaccurate, or insufficient to meet the burden of evidence.
**Stated pitfall(s)**	Collection, processing, and comparison of DNA samples from multiple individuals can carry logistical, temporal, geographical, and financial burdens. The government might not collect samples or request or apply DNA testing results uniformly in decision making processes.
**Narrative**[4]	A heterosexual married American couple is in the process of adopting two-year-old twins from a West African country. In 2018 they were living in the twins' country of origin; they were given custody of the children in the summer of 2018, and the adoption was finalized on the part of the West African country's government by the end of 2018. The couple began the U.S. adoption process at the beginning of 2019 and were invited to apply for adoption visas at the beginning of 2020 to return to the United States with their children. At their visa interview at the U.S. embassy located in a second West African country, however, immigration officials told the couple that they needed to provide more evidence. They began to gather materials, but border closures and travel restrictions due to the coronavirus pandemic made gathering all the required evidence impossible. Documents from the twins' country of origin are considered unreliable, so DNA evidence of the relationship between the birth parents and the twins would be helpful. The family now lives in the second West African country where their interview was held. One of the birth parents lives in the twins' country of origin; the U.S. embassy in the twin's country of origin will not cooperate to DNA test this birth parent. The family wishes to return to the United States due to safety concerns amid the pandemic, but cannot do so with the twins, who are not U.S. citizens. They, along with other families in similar positions, have petitioned the U.S. government to grant emergency exceptions for visas amid the pandemic.

[4] A Westerman. 'We'd Have to Abandon Our Daughters:' Pandemic Delays International Adoptions. *NPR* (14 April 2020). Available at https://www.npr.org/2020/04/14/831893954/we-d-have-to-abandon-our-daughters-pandemic-delays-international-adoptions

CONTEXT 4: DNA testing for verification of parent-child relationships at border entry points

Case 25: DNA testing request reveals fraud

**Source**	Study team reported
**Region**	Central America
**DNA test type**	Rapid DNA
**Relationship tested**	Father-child
**Interpreted value**	DNA testing requests or requirements can deter intentionally fraudulent misrepresentations of relationships or clarify misunderstandings around the kinds of relationships that qualify for immigration benefits.
**Interpreted pitfall(s)**	No pitfalls interpreted
**Narrative**[5]	A Central American man was encountered by Homeland Security Investigation agents in Texas. He was with an infant child that he initially claimed was his. While he was being interviewed for potential rapid DNA testing, he confessed that he had presented a fraudulent birth certificate for the infant child, who was not related to him. He had reportedly "purchased" the infant shortly after birth from the birth mother for the equivalent of approximately $84. The man did not present any documents to validate either a legal adoption of the infant or documents from the birth mother indicating her consent to legally transfer custody of the infant. The subject further admitted the infant was purchased solely as a means for him to enter the United States. The man was charged with violations of alien smuggling and illegal entry.

[5] Nevano, G.C. Statement of Gregory C. Nevano, Assistant Director, Investigative Programs, Homeland Security Investigations. U.S. Dpeartment of Homeland Security (26 June 2019). Available at https://www.hsgac.senate.gov/imo/media/doc/Testimony-Nevano-2019-06-26.pdf

CONTEXT 5: Comparison of family reference samples to unidentified human remains samples for identification purposes in transnational missing persons cases

Case 26: DNA testing documents a genetic relationship

**Source**	Study team reported
**Region**	Central America
**DNA test type**	STR typing at a forensic laboratory
**Relationship tested**	first-degree relative
**Interpreted value**	DNA testing can provide documentation of genetic relationships and identity when other forms of documentation are unavailable, inaccurate, or insufficient to meet the burden of evidence.
**Interpreted pitfall(s)**	No pitfalls interpreted
**Narrative**[6]	A woman left for the United States after her husband had been killed in her home country. She intended to join family in New York, find work, and send money back home. She crossed the U.S.-Mexico border in Texas; two days after she crossed, a deceased, unidentified woman was found in a county 50 miles north of the border. The remains of the woman were interred in a cemetery with a temporary marker that read "Unknown Female, [location where body was found]." Years later, a team of forensic anthropologists exhumed her remains as part of their efforts to identify transnational missing persons. They found among her remains, in the insoles of her shoes, an identification card bearing her name. This led the team to search NamUs for a corresponding missing persons' report. A report was found in NamUs, but no DNA sample had been obtained. Her family in her home country was contacted by an NGO who collected DNA samples from biological relatives. Two weeks later, a DNA association report, along with a comparison of antemortem and postmortem data, suggested that the remains did belong to the woman named on the ID card she was carrying.

Case 27: DNA testing documents a relationship, but is rejected by the family

**Source**	Study team
**Region**	Central America
**DNA test type**	STR typing at a forensic laboratory
**Relationship tested**	First-degree relative

[6] Rose, A. The forensics of identifying migrants who die exhausted after crossing from Mexico. in *Scientific American* (2015). Available at https://www.scientificamerican.com/article/the-forensics-of-identifying-migrants-who-die-exhausted-after-crossing-from-mexico/

**Interpreted value**	DNA testing can provide documentation of genetic relationships and identity when other forms of documentation are unavailable, inaccurate, or insufficient to meet the burden of evidence.
**Interpreted pitfall(s)**	Kinship analysis can reveal sensitive information. Collection, processing, and comparison of DNA samples from multiple individuals can carry logistical, temporal, geographical, and financial burdens. The government might not collect samples or request or apply DNA testing results uniformly in decision-making processes.
**Narrative**	Skeletal remains were found on private land in Texas. Near the remains, an identification card and passport with a name were discovered. Law enforcement notified the Justice of the Peace and the local funeral home buried the remains with a temporary marker using the name on the passport. However, no DNA specimen was submitted for genetic typing and comparison to a family reference sample prior to burial as required by state law. Furthermore, no official identification by a legal jurisdictional authority was performed; rather, an assumption was made that the remains were associated with the name on the passport. Because there had been no official identification, legally, the remains were classified as unidentified. Seven years after the remains were found, a team of forensic anthropologists exhumed them as part of their efforts to identify transnational missing persons. A DNA specimen was submitted to a government-funded laboratory for comparison to family reference samples collected by a consul. Notification of a genetic association was received almost a year later based on a partial STR profile. To date, the woman identified as the mother of the deceased via genetic association does not accept the identification as she believes she saw a photo of her child in a detention center after they were reported missing.

Case 28: Infrastructure delays or prevents a DNA identification

**Source**	Study team communication
**Region**	Central America
**DNA test type**	STR typing at a forensic laboratory and SNP typing at a commercial laboratory
**Relationship tested**	first-degree relative and distant kinship
**Interpreted value**	DNA testing can provide documentation of genetic relationships and identity when other forms of documentation are unavailable, inaccurate, or insufficient to meet the burden of evidence.
**Interpreted pitfall(s)**	Collection, processing, and comparison of DNA samples from multiple individuals can carry logistical, temporal, geographical, and financial burdens. DNA from the appropriate individuals to test a relationship is not always available. The appropriate technology and/or infrastructure to test a relationship is not always available.

**Narrative**	The body of an unidentified man was recovered and sent to a medical examiner for identification. The medical examiner estimated that the man was between 30-45 years old and had been deceased for several months at the time of recovery. Along with his remains was a scrap of paper with names and phone numbers and a photograph of a woman. Humanitarian groups have been unsuccessful investigating the phone numbers and photograph but found clues that the individual might be from a specific Central American country and that the photograph might be of the mother of his child. His remains were genotyped for STRs, but no kinship matches have resulted to date. It is possible that his family provided family reference samples to a database that is not being compared to the one that holds his data or that the family has not come forward to provide family reference samples. His DNA sample was also genotyped for SNP data in order to cross-reference his data with potential relatives' data in genealogical databases. Distant relatives were theorized from the data, but no identifications have been made to date. However, ancestry informative markers resulting from the SNP data provided more evidence that the he could be from the country identified by humanitarian groups.

Case 29: Infrastructure delays or prevents a DNA identification

**Source**	Study team communication
**Region**	Unknown
**DNA test type**	STR typing at a forensic laboratory
**Relationship tested**	First-degree relative
**Interpreted value**	DNA testing can provide documentation of genetic relationships and identity when other forms of documentation are unavailable, inaccurate, or insufficient to meet the burden of evidence.
**Interpreted pitfall(s)**	DNA from the appropriate individuals to test a relationship is not always available. The appropriate technology and/or infrastructure to test a relationship is not always available. The government might not collect samples or request or apply DNA testing results uniformly in decision-making processes.
**Narrative**[7]	As part of their efforts to identify transnational missing persons, a team of forensic anthropologists had plans to exhume at least eight unidentified individuals from eight burial sites in a cemetery based on the temporary grave markers that were still visible. They returned from the cemetery with 28 body bags, the majority from unmarked burial sites discovered by talking with local community members. One of the bodies was an unidentified individual who had been found on private land in Texas 5 years prior to exhumation. Contrary to state law, no DNA specimen had been collected prior to burial. A DNA

[7] Matthews, J.D. OP-ID. 32 minutes (2018). Available at https://filmfreeway.com/OP-ID

	specimen was submitted to a government-funded laboratory about a few months after exhumation for typing. Notification that a full profile was obtained and uploaded to the federal database known as the Combined DNA Index System [CODIS] was received approximately nine months later. To date, no family reference samples have matched. It is possible that the family of the deceased provided family reference samples to a database that is not being compared to the unidentified human remains index in CODIS or that the family has not come forward to provide family reference samples.

Case 30: Infrastructure delays or prevents a DNA identification

**Source**	Study team communication
**Region**	Unspecified
**DNA test type**	STR typing at a forensic laboratory
**Relationship tested**	First-degree relative
**Interpreted value**	No utility interpreted
**Interpreted pitfall(s)**	The appropriate technology and/or infrastructure to test a relationship is not always available. The government might not collect samples or request or apply DNA testing results uniformly in decision-making processes.
**Narrative**	A person crossed into Texas from Mexico with a group led by a coyote (a paid escort) over five years ago. The coyote escorting the group dropped them off at a location in Texas and told them to walk north to bypass a U.S. Customs and Border Protections checkpoint. Customs and Border Protection [CBP] agents spotted the group traveling, and a chase ensued that resulted in some migrants being captured and others scattering as they ran away. The person in question was among those who scattered and has not been heard from since. Periodic search and recovery efforts led by the county where the person disappeared and humanitarian groups have not yielded any sign of them. The families provided DNA family reference samples, assuming the worst has happened. Thus far, there have been no reports of a match to unidentified human remains. It is possible that the body has not been found or that the DNA data from the human remains has not been provided to a database for comparison to the data from the provided family reference samples.

Case 31: Officials will not collect a DNA sample

**Source**	Study team reported
**Region**	Unknown
**DNA test type**	STR typing at a forensic laboratory
**Relationship tested**	First-degree relative

**Interpreted value**	No utility interpreted
**Interpreted pitfall(s)**	The government might not collect samples or request or apply DNA testing results uniformly in decision-making processes.
**Narrative**[8]	During the coronavirus pandemic, Border Patrol agents found a person who was dehydrated and semi-responsive in Texas. The person was taken to a Texas hospital where they were sedated and placed on a ventilator. They tested positive for COVID-19 and died a few days later. They were not carrying any identification and CBP did not find a match for their fingerprints in their system. The person's remains could not legally be buried or cremated prior to DNA sampling, but the hospital would not collect DNA samples without an autopsy and would not preform autopsies on COVID-positive patients. The remains were moved to a separate, refrigerated trailer while authorities determined how to identify the person so the family could be notified and the remains returned to them. No further information on the case has been reported.

[8] Gonzalez, V. Death of unidentified migrant who died in federal custody after testing positive for COVID-19 highlights complex state process. *KRGV.com* (10 July 2020). Available at https://www.krgv.com/news/death-of-unidentified-migrant-who-died-in-federal-custody-after-testing-positive-for-covid-19-highlights-complex-state-process/
