# Supplementary material for: Framing the utility and potential pitfalls of relationship and identity DNA testing across United States immigration contexts
Source: HGG Adv. 2021 Sep 24;3(1):100060. doi: 10.1016/j.xhgg.2021.100060 (PMC8756547; doi:10.1016/j.xhgg.2021.100060)
Supplement: Document S2. Article plus supplemental information [file mmc2.pdf]

HGG Advances

ARTICLE

Framing the utility and potential pitfalls of relationship and identity DNA testing across United States immigration contexts

Diana Madden,[1] Brianna A. Baker,[2] Jennifer K. Wagner,[3] and Sara H. Katsanis[1,4,*]

Summary

Genetic information is increasingly used at US border entry points, but the use of DNA in immigration contexts is not new. DNA testing for verification of identity or relationships for visa and asylum petitions began in the 1980s. Long-standing applications demonstrate both the utility and pitfalls of DNA testing in immigration contexts. Some of these pitfalls are shared with health-related contexts of DNA testing, but the power of government officials to deny immigration benefits, separate families, or make accusations of fraud among a vulnerable population elevates the potential harms, including stigmatization, discrimination, and coerced consent. We conducted semi-structured interviews with professional stakeholders on their understandings of the process of DNA testing, opinions on the role of DNA testing in immigration, and experiences with DNA applications in immigration. From the 22 interviews, we sourced 21 case examples involving DNA testing and supplemented these with 10 case examples provided by the study team. The 31 case examples capture instances of DNA testing for relationship or identity across five immigration contexts. Using the case examples, we developed three overarching utilities and six overarching pitfalls of DNA testing that apply across these immigration contexts. Our framework allows long-standing applications of DNA testing in immigration to inform stakeholders' approaches to applications in new contexts. As the use of DNA data in immigration contexts expands, its implementation should recognize the utility of DNA data to both migrants and government while guarding against pitfalls that could undermine the human rights and dignity of a vulnerable population.

Introduction

The ethical and social implications of DNA relationship testing in the context of immigration are compounded by a combination of the vulnerabilities of migrant populations and the potential harms that might arise from testing.[1–5] Many of the potential harms of DNA testing are shared across immigration and health-related contexts, including discrimination, stigmatization, privacy violations, revelation of sensitive information (such as misattributed parentage), and poorly informed or coerced consent. In immigration contexts, these risks are heightened by the power differential between those undergoing testing and those ordering the tests. While healthcare providers might be considered figures of authority, immigration agents or officials have the power to make decisions about families' futures based on genetic information. The recent, rapid expansions of DNA testing for relationship verification in US immigration contexts—in both volume and purpose—demonstrate this power.

DNA data have been used to verify relationships for family-based immigration visas since a 1985 case in the United Kingdom.[6] In the United States, DNA testing was first harnessed as evidence for family-based immigration visas in a legacy Immigration and Naturalization Services (INS) memo in 2000, which instructed that officers could suggest DNA testing in cases where other forms of documentation failed to verify a relationship.[7] DNA testing has continued to be voluntary in most cases, with few exceptions (Table 1). Where DNA testing is voluntary, it might be requested by US Citizenship and Immigration Services (USCIS) after reviewing the evidence of the relationship provided; attorneys and/or clients also may choose to use DNA testing if they know there is little other documentation (e.g., birth certificate) of a relationship. Commercial relationship laboratories provide DNA testing for family-based immigration visas; currently, relationship testing laboratories must be accredited by the American Academy of Blood Banks (AABB) for results to be accepted in immigration and legal proceedings.[1] Generally, only close relationships, such as parent-child or siblings, are tested. The genetic markers tested depend on the laboratory, but most use a set of 20–30 standardized, highly polymorphic short tandem repeat (STR) markers. The laboratory will issue a report indicating the likelihood of the tested relationship to the ordering party and the government agency seeking the results.[1]

Family-based DNA testing for the identification of people who die crossing the US-Mexico border is another long-standing use of DNA in an immigration context.[29] Logistical, legal, and ethical challenges plague the DNA data sharing in missing migrants' cases.[31] Families may

[1]Mary Ann & J. Milburn Smith Child Health Outcomes, Research, and Evaluation Center, Ann & Robert H. Lurie Children's Hospital of Chicago, Chicago, IL 60611-2991, USA; [2]Stigma, Identity, and Intersectionality Research Lab, Teachers College, Columbia University, New York City, NY 10027, USA; [3]School of Engineering Design, Technology, and Professional Programs, Pennsylvania State University, University Park, PA 16802, USA; [4]Department of Pediatrics, Feinberg School of Medicine, Northwestern University, Chicago, IL 60611-2991, USA

*Correspondence: skatsanis@luriechildrens.org

https://doi.org/10.1016/j.xhgg.2021.100060.

© 2021 The Author(s). This is an open access article under the CC BY license (http://creativecommons.org/licenses/by/4.0/).

Table 1. Contexts for relationship DNA testing in US immigration

Context	Background	Current status of DNA testing
Visa petition context: identity verification for visa or citizenship applications and petitions for non-citizen relatives	Relationship DNA testing was formally introduced to the US immigration system in 2000.[7] In 2008, DNA testing was piloted among refugees for P-3 family reunification; fraud was reportedly revealed in East Africa,[8] and the program was suspended.[9] In 2012, the P-3 program resumed with a requirement for DNA testing,[9] but it became defunct under the 2017 "Muslim ban."[10] Under the Obama administration, DNA testing was required to verify the parentage of petitioning Central American minors (CAM);[11,12] the CAM program was closed during the Trump administration.[13]	Relationship DNA testing is conducted by AABB-accredited laboratories,[14] most of which are commercial. Close relatives (e.g., parents, siblings) of petitioners might be asked to complete a DNA test in support of a relationship claim for, for instance, a Petition for Alien Relative (I-130) or Refugee/Asylee Relative Petition (I-730). Relationship DNA testing might also be used to verify identity and qualification for citizenship for an Application for Certification for Citizenship (N-600). DNA testing remains voluntary for most petitions but is required for the P-3 and CAM programs, to the extent that they are operational.
Unaccompanied youth context: relationship verification for placement with sponsors of unaccompanied migrant youth	Unaccompanied youth refers to children under 18 years of age who enter the United States without a parent or legal guardian or children who are separated upon entry from their adult caregiver.[15] Since 2003, ORR has been responsible for the care and resettlement of UC, which includes determining the most suitable placement for a child.[16,17]	ORR states that "DNA matching is often used in the ORR UC program when documents are not available or unverifiable,"[18] but the details of this use are unclear. In January 2021, ORR proposed revisions to two of the forms used to assess the suitability of a sponsor for a child; the proposed revisions include a change to form SVP-3/3 s that would allow sponsors "to voluntarily submit to a DNA test to prove they are biologically related to the child…in lieu of supporting paperwork."[19]
Government separation context: verification of parent-child relationships following government-imposed family separation	The "zero-tolerance" policy was ended by executive order on June 20, 2018.[20] Days later, a US District Judge ordered the reunification of migrant families within 14–30 days.[21] HHS announced that it would use DNA tests to verify parent-child pairs in lieu of other forms of documentation to speed the process.[22] Ongoing investigations have since revealed that the time frame and scale of separations were more extensive than previously thought.	As of June 2021, it is estimated by the Biden administration's Interagency Task Force on the Reunification of Families that 2,127 children have not yet been reunified with their parent(s). The task force continues to identify instances of separation.[23] Of the children thought to remain separated, it has not yet been possible to contact the parents, guardians, or attorneys of 368.[24] These families could benefit from DNA-led reconnections outside of government control.[25]
Family verification context: verification of parent-child relationships at border entry points	In May 2019, the DHS conducted a pilot program at select border checkpoints over several months, motivated by reports of an increase in family unit fraud; the pilot was later extended from June to November 2019.[26] Rapid DNA testing was used on site to verify claimed biological parent-child relationships in family units suspected of fraud.[20]	Over 5 months in 2019, the rapid DNA testing did not verify 24.7% of family units tested.[27] The extent of the rapid DNA testing beyond 2019 is unknown; however, the 2020 biometrics proposed rule[27] that was rescinded by the Biden administration[28] proposed sustaining rapid DNA testing at the border.
Transnational missing context: comparison of FRSs to UHR samples for identification purposes in transnational missing persons cases	DNA from UHR is compared to FRSs to aid in identifications.[29] Samples taken by US law enforcement can be uploaded to the CODIS, the federal DNA database of the United States; samples taken by NGOs are sent to private laboratories.[29]	Not all migrant remains are found, and those who are found might be buried or cremated before DNA is taken.[30] Few families of missing migrants come forward to provide FRSs, possibly out of fear of law enforcement; others might approach an NGO to provide DNA. UHR samples and FRSs might be sent to different systems depending on the involvement of law enforcement and various NGOs in collection, resulting in silos of UHR and FRS data that cannot be compared.[31]

Abbreviations: P-3, Priority 3 refugee program; CAM, Central American Migrant Minors Program; AABB, American Association of Blood Banks; ORR, Office of Refugee Resettlement; UC, unaccompanied children; HHS, US Department of Health and Human Services; DHS, US Department of Homeland Security; UHR, unidentified human remains; FRS, family reference sample; CODIS, Combined DNA Index System; NGO, non-governmental organization.

provide family reference samples (FRSs) for comparison to DNA samples from unidentified remains. For migrant families, families may choose to provide DNA FRSs to non-governmental organizations (NGOs) for comparison in a private database or to law enforcement for comparison to the federal DNA database, the Combined DNA Index System (CODIS). Any family member can provide an FRS, although ideally multiple close relatives, particularly maternal relatives, will provide samples. FRSs are typed for the 20 STRs commonly tested in forensic casework and that comprise the data in CODIS. FRSs in CODIS are not compared to the criminal evidence index, only to the unidentified remains index. While DNA identification of human remains can be successful, it is hampered by the fragmentation of unidentified human remains (UHR) and FRSs into different databases with policies preventing DNA data sharing.[2]

Beginning in 2018, new immigration contexts for the use of DNA testing gained the attention of both policymakers and the public. In April 2018, under the "zero-tolerance" immigration policy,[32] migrant children were separated from their caregivers;[33] soon after the practice

was ended, the US Department of Health and Human Services (HHS) announced plans to use DNA to help reunify the separated families.[34] Some of us authors tracked the extensive discussion by the media, policymakers, and the public of this potential DNA application.[35] In May 2019, amid an increase in families arriving at the US-Mexico border, US Immigration and Customs Enforcement (ICE) began piloting the use of rapid DNA instruments at select border checkpoints to verify parentage claims of family unit pairs.[20] Unlike DNA testing for family-based immigration visas, family verification testing at border sites is done on site using rapid DNA instruments. Currently, rapid DNA instruments test for STRs, and the policy indicates only parent-child relationships are tested.[26] Families are selected for testing by government agents; testing is voluntary, but refusal might affect a family's immigration status. In September 2020, DHS appeared to be poised to expand the scope of this rapid DNA testing with a Notice of Proposed Rule Making (NPRM) on Collection and Use of Biometrics by USCIS.[27,36] The NPRM has since been retracted under the Biden administration.[28] As 2021 progresses, the number of families and unaccompanied migrant youth arriving at the southern US border is rising steeply,[37] and new bills have been introduced that suggest DNA testing to detect child trafficking.[38] Several of us authors are part of efforts by the DNA Bridge consortium to develop trauma-informed processes and international, non-governmental structures to allow families to use DNA relationship testing to aid in locating and reunifying with their children.[25]

These recent expansions of DNA testing in both volume and scope, as well as continued attention from policymakers and the public, call for a framework for developing cross-context guidelines informed by long-standing uses of DNA data. Such guidelines should be grounded in an understanding of the utility of DNA tests as they pertain to measuring family relationships and the pitfalls that can emerge. We selected the terms "utility" and "pitfall" over familiar contrasting pairings such as pro/con, benefit/risk, or advantage/disadvantage, as each of these sets of terms implies a self-directed choice made by the person to pursue a DNA test. We borrow the term utility from the use of "clinical utility," in that it captures the practical value of a test on health outcomes.[39,40] The power dynamics, test type, and testing processes in the immigration context differ from a clinical context, so we have adapted the meaning of utility accordingly. Utility in an immigration context might include personal utility to an individual,[40,41] institutional utility (i.e., to the immigration system), or societal utility. Our utility/pitfall framework encompasses both the benefits/risks to the person and the utilities/pitfalls of the DNA test within an immigration process. For instance, we can frame the utility of DNA test results as evidence in an immigration case to the petitioner and to the government, and we can frame the pitfall of the unexpected challenges or burdens arising from DNA testing processes in an immigration context.

To determine what the pitfalls and utilities of DNA relationship testing in immigration contexts might be, we conducted semi-structured interviews with key stakeholders involved with immigration and/or DNA testing in immigration contexts. We consider this framework over the course of the DNA testing process, including pre-testing processes such as requests for DNA testing and consent, sample collection and analysis, communication of results, and role of results in determining case outcomes. We outline the five currently relevant immigration contexts where DNA relationship testing might be used (Table 1) to provide clarity as to how the utility/pitfall framework applies across contexts. The recent expansion of DNA collection from migrant detainees for criminal justice purposes is an instance of the use of DNA data in an immigration context;[42] in this context, however, DNA data are collected and held for criminal or missing persons investigations, not with the intention of conducting kinship analysis. Ancestry DNA testing also has been used in an immigration context to support or investigate nationality claims.[43] We exclude both of these applications from our analysis, as our framework is tailored specifically to relationship DNA testing in immigration.[43]

Using a body of case examples sourced from our interviews and supplemented with case examples provided by the study team, we lay out a cross-context framework that captures the utilities and pitfalls of relationship DNA testing in immigration. As immigration to the United States continues to expand and new technologies, policies, and programs emerge, our framework based on factual case examples can be mapped onto new and shifting contexts to mitigate accumulation of harms.

Subjects and methods

Human subject protections

This study was conducted under Duke University Institutional Review Board (IRB) #2018-0510 and Lurie Children's Hospital IRB #2019-2909. A consent information sheet was provided to participants prior to the interview, and verbal consent for audio recording was taken at the start of the interview. Permission for re-contact was also recorded. An NIH Certificate of Confidentiality covered the protocols and data collected from participants.

Participant recruitment

We used information-oriented sampling to identify professional stakeholders involved with immigration processes who might know of or encounter DNA testing in their work, including immigration attorneys, NGO leadership and attorneys, technology company officials, academics, journalists, and representatives of government, law enforcement, and medicolegal agencies. We then compiled a further list of immigration attorneys from the American Immigration Lawyers Association website for random sampling, selecting for representation of geographical regions of the United States. We opted to speak with professional stakeholders, not immigrants or migrants and their families, because we anticipated that professional stakeholders would have a more varied experience of DNA testing in their fields. Additionally, we

believed that the understanding and actions of the group of stakeholders we identified would have direct implications for immigrants or migrants and their families. This approach allowed us to explore how DNA testing plays out on the ground without interacting with potentially vulnerable groups. Recruitment emails were sent to candidate participants soliciting a reply of interest. We targeted 20–30 interviews with 2–3 participants from each stakeholder category, oversampling for immigration attorneys to gather diverse experiences.

Semi-structured interviews

One semi-structured interview guide was used for all participants, regardless of profession. The guide focused on gathering data on the following: (1) knowledge of how DNA tests are used in immigration; (2) experience working with migrants, migrant families, and DNA testing; (3) basic comprehension of the purpose and process of DNA testing; and (4) opinions of the risks and benefits of DNA testing in immigration contexts. In addition, one Likert-scale question on the importance of DNA testing in immigration was initially included and later adapted into an open-ended question due to the struggle of participants to express their views via a numerical scale. Interviews were conducted in person or by phone between February and June 2019 and audio recorded to enable transcriptions.

Interview coding

Interview transcripts were checked by two study team members, including one interviewer, for accuracy. Transcripts were then coded to capture: (1) applications of DNA testing in immigration contexts, (2) scope of participant experiences and knowledge of DNA testing, and (3) participant perspectives on general benefits and risks of DNA testing in immigration. Interviews were further coded for any mention of specific cases involving DNA testing, descriptions of the types of situations where DNA testing is used, and descriptions of DNA testing processes. Each case, use instance, and process description was summarized from participant statements and collated into a spreadsheet.

Coders then generated a set of pitfalls that emerged as themes across all interviews. While the pitfalls emerged from descriptions of cases, use instances, and processes, descriptions of specific case examples best illustrated these pitfalls. Case examples were then coded with additional categories: case type, immigration benefit sought, relevant biological/legal/social relationships, case status, number of people involved and their location(s), details of DNA test type if any, who requested testing, who was tested, instance of fraud (yes/no [Y/N]), and instance of misattributed parentage (Y/N).

Development of case examples

Participant descriptions of cases with a high level of contextual detail were selected for development into case examples. Our aim was to capture the broadest possible range of actual uses of DNA testing in an immigration context. The summaries of the case examples from the initial coding process were refined into narrative form (Supplemental notes). The coding of the case type was used to assign each case example to one of the five immigration contexts (Table 1). Case examples were grouped by immigration context and titled with a brief description of the exemplified pitfall(s) or utility(ies). Participants who provided certain cases that were missing key details needed to develop the narrative consistently across examples were re-contacted for additional information, and their responses were incorporated into the specific case example narrative.

The case example narratives were reviewed by two coders to check for accuracy and clarity. Interview transcripts were referenced where necessary to verify that the participant's meaning and the level of detail provided were preserved in the narrative. A third coder read the final case examples for clarity and accessibility to audiences of diverse expertise.

Parallel to the development of case examples from interviews, study team members were asked to share cases within the five immigration contexts that came to their attention. Most of these cases were already in the public domain in academic, governmental, or media reports; these were anonymized, coded, and developed into narratives.

Development of pitfalls and utilities

Case examples were grouped according to similarities between the pitfalls and/or utilities of DNA testing that they demonstrated. Case examples could belong to more than one group. A summary statement of the utility(ies) or pitfall(s) demonstrated by each group was crafted to capture the elements that applied across cases and immigration contexts. Coders reviewed the applicability of each pitfall to each immigration context. We requested the input of the study team on the immigration contexts framework and integrated their feedback. Finally, two independent coders re-coded the case examples to ensure that they fit within the overarching framework.

Results

Participants

A total of 181 professionals were identified for potential contact: 125 immigration attorneys, 33 NGO representatives, nine government or law enforcement officials, nine academics, and five technology company representatives. Invitation and follow-up invitation emails were sent to all but six of these stakeholders. Of the 175 invitations, 28 responded (response rate of 16%), and 23 (13%) agreed to interviews. The 23 participants included 13 immigration attorneys, five NGO representatives, two academics, and three technology company representatives; participants are represented here via codes that reflect their profession (Table 1). TC08 and TC09 were interviewed together. One participant declined audio recording; all agreed to re-contact.

Participant experience with DNA testing in immigration contexts

Participants' interactions with immigrants and/or migrants and experience with DNA testing in immigration contexts varied by profession and in some cases between participants. Overall, the experiences of the participants slanted toward family-based applications for US visas (or petitions by refugees or asylees). The companies that the three technology company representatives (TC07, TC08, TC09) worked with specialized in immigration-related DNA testing. Both of the academics (AC03, AC05) and the three NGO directors (NG02, NG06, NG14) had limited

direct interaction with immigrants and/or migrants but engaged as supervisors or through their professional work with multiple contexts where DNA testing might occur. The two NGO attorneys (NG15, NG21) worked primarily on family-based applications for refugees and asylees and sponsorships for unaccompanied minor children. Most of the immigration attorneys' encounters with DNA testing involved visa or citizenship applications and petitions for noncitizen relatives.

Of the 11 participants who gave an explicit characterization of how often they encountered DNA testing in their work with immigrants and/or migrants, 10 stated that they encountered DNA testing infrequently; for instance, IA01 summarized, "maybe I'll have one [instance of DNA testing], maybe two a year." Only one participant, IA10, reported frequent DNA testing, stating, "Yes, actually, I—we see it quite a bit and in fact I feel like the practice of requesting DNA test—tests has really gone up in the last 10 years or so where you can expect it on, you know, a good percentage of the cases."

Despite overall infrequent experience of DNA testing, participants emphasized its importance in immigration contexts. Participants IA01, NG02, AC05, and IA04 struggled to assign a number to the importance of DNA testing in immigration on a Likert scale (with "5" being very important). IA01 captured the tone of the ambiguity, saying, "No, I mean, I think on the cases where it is used, it's probably a five because it's really important in those specific cases to be able to have some type of biological match. But it's just, it's not something that we commonly use." In lieu of the Likert-scale question, the remaining participants were asked how much weight is given to DNA testing in immigration contexts, which prompted wider-ranging considerations of the utility of DNA to different stakeholders (attorneys, clients, or the government). Participants particularly emphasized the ability of positive or negative results of DNA testing to determine the outcome of a case: for instance, IA10 stated, "Yeah, I mean, well, it's helpful if it's a match. And it's incredibly unhelpful if it's not a match. You know? …if the government is requesting a DNA test, um, whether it's positive or negative will be the determining factor in the case." NG02 emphasized the value placed by the government on DNA test results as "clear objective evidence" of a relationship.

Case examples

Coding of the interviews yielded 45 cases, 42 use instances, and 19 descriptions of processes. Of the 45 cases, 21 were developed into case example narratives (Table 2). The 24 cases not selected for narrative development were similar in content to cases for which more detail was provided. An additional 10 cases were sourced from the study team, of which seven are in the public domain. The full catalog of case examples, referenced here by case number, is available as Supplemental notes. No case examples were sourced from nine of the participants. Four case examples were sourced from NG06, with one to two case examples sourced from each of the remaining participants. Twenty-two of the 31 total cases fell under the visa petition context, including all but three of the case examples sourced from participants. The transnational missing context had six case examples, all of which were supplied by the study team.

Utilities and pitfalls of DNA testing in immigration contexts

We identified three overarching utilities and six overarching pitfalls that apply to DNA testing for relationship verification across the five immigration contexts. The three utilities are: (A) DNA testing can provide documentation of genetic relationships when other forms of documentation are unavailable, inaccurate, or insufficient to meet the burden of evidence; (B) DNA testing can disprove or detect fraud when claimed relationships or identity are in question; and (C) DNA testing requests or requirements can deter intentionally fraudulent misrepresentations of relationships or clarify misunderstandings around the kinds of relationships that qualify for immigration benefits. The six pitfalls are: (A) Family is not defined by genetic relationships alone; kinship terms do not correspond to biological (or genetic) relationships in the same way across languages and cultures; (B) kinship analysis can reveal sensitive information; (C) collection, processing, and comparison of DNA samples from multiple individuals can carry logistical, temporal, geographical, and financial burdens; (D) DNA from the appropriate individuals to test a relationship is not always available; (E) the appropriate technology and/or infrastructure to test a relationship is not always available; and (F) the government might not collect samples or request or apply DNA testing results uniformly in decision-making processes. Case examples often demonstrated both utilities and pitfalls. Of the 31 cases, 11 were coded for multiple pitfalls, and 12 were coded for both a utility and at least one pitfall. Table 3 shows case examples by utility and immigration context, and Table 4 shows case examples by pitfalls and immigration context.

Utility A: DNA testing can provide documentation of genetic relationships when other forms of documentation are unavailable, inaccurate, or insufficient to meet the burden of evidence

Participants named lack of documentation as a primary reason DNA testing is used for visa or citizenship applications and petitions for noncitizen relatives; often, DNA testing ultimately supported access to an immigration benefit by providing documentation of relationships. Participants often described challenges presented by birth certificates. In case 01 and case 18, DNA testing provided genetic documentation of a father-child relationship where the father was not listed on the birth certificate. In case 02, the grandparents had chosen to be listed as the parents on the birth certificate of their grandchild, whom their young daughter had out of wedlock; DNA testing provided genetic documentation of a mother-child relationship for their daughter and grandchild's visa petition. In case 13, the birth certificate correctly listed the relevant

Table 2. Characteristics of case example sources

Participant or source	Profession	Gender	Visa petition context	Unaccompanied youth context	Government separation context	Family verification context	Transnational missing context
IA01	immigration attorney	M	cases 8, 9				
IA04	immigration attorney	F	case 2				
IA10	immigration attorney	F					
IA11	immigration attorney	M	case 4				
IA12	immigration attorney	M					
IA13	immigration attorney	M	case 18				
IA16	immigration attorney	F		case 20			
IA17	immigration attorney	F	cases 13, 16				
IA18	immigration attorney	F	case 7				
IA19	immigration attorney	M	case 1				
IA20	immigration attorney	M					
IA22	immigration attorney	F					
IA23	immigration attorney	F	case 14				
NG02	NGO director	M			case 21		
NG06	NGO policy director	F	case 15	case 12			
NG14	NGO director	F					
NG15	NGO attorney	F	cases 11, 17				
NG21	NGO attorney	F	cases 3, 10, 19				
AC03	academic	M					
AC05	academic	F					
TC07	technology company representative	M	case 6				
TC08 & TC09	technology company representative	M, F	case 5				
Study team		N/A	cases 22, 23, 24			case 25	cases 26–31

See Table 1 for context descriptions. M, male; F, female; N/A, not applicable.

father-child relationship, but the certificate's authenticity was questioned; DNA testing provided documentation. In case 14, a client had been deported several times despite a birth certificate naming a US citizen as his father; the client and attorney hoped that DNA testing would demonstrate the paternal relationship to support a claim to citizenship, although testing had not been completed at the time of the interview.

Participants also described cases where the absence of a history of interactions or a caregiving relationship was the primary barrier to the desired immigration benefit. For example, describing an as-yet-unresolved case (case 17), NG15 questioned the potential usefulness of DNA testing as documentation to support the reunification of a father and child estranged due to war, given the lack of a caregiving relationship. In case 18, DNA documentation initially was considered insufficient to support the reunification of a father and child with no history of a caregiving relationship, but the initial denial was ultimately overturned on the basis of the genetic relationship.

Documentation or clues to identify and connect transnational missing persons to families also can be scarce, making DNA testing a valuable tool. DNA testing in combination with other evidence in case 26 allowed for the identification of a person whose family had been searching for her. But DNA testing does not always provide resolution. In cases 28 and 29, forensic anthropologists submitted DNA samples from UHR for upload to CODIS, but to date no FRSs have matched.

Utility B: DNA testing can disprove or detect fraud when claimed relationships or identity are in question

In some case examples, government suspicion was directed at those seeking an immigration benefit, and DNA testing helped remove suspicion. In case 04, a DNA test was ordered by a client and his attorney to provide evidence of the client's identity, relieving the government's suspicion that he was not eligible for naturalization. In case 23, a man

Table 3. Utilities of relationship and identity DNA testing across US immigration contexts

	Visa petition context	Unaccompanied youth context	Government separation context	Family verification context	Transnational missing context
No utility stated or interpreted	cases 9–11 12, 15, 19, 22[a]	no case examples from study team; case 20	no case examples from study team; case 21	no case examples	no case examples from interviews; cases 30,[a] 31[a]
Utility A: DNA testing can provide documentation of genetic relationships and identity when other forms of documentation are unavailable, inaccurate, or insufficient to meet the burden of evidence.	cases 1–3, 13, 14, 16–18, 24[a]	no case examples	no case examples	no case examples	no case examples from interviews; cases 26–29[a]
Utility B: DNA testing can disprove or detect fraud when claimed relationships or identity are in question.	cases 4–6, 08, 23[a]	no case examples	no case examples	no case examples	no case examples
Utility C: DNA testing requests or requirements can deter intentionally fraudulent misrepresentations of relationships or clarify misunderstandings around the kinds of relationships that qualify for immigration benefits.	no case examples from study team; case 7	no case examples	no case examples	no case examples from interviews; case 25[a]	no case examples

See Table 1 for context descriptions.
[a]Case examples sourced from the study team, not from interviews.

who was born to two US citizens and adopted abroad was in danger of being deported from the United States; he hoped DNA testing, in conjunction with other evidence, would demonstrate his claim to US citizenship. In case 08, it was the client and her attorney who used a DNA test to verify a claim. The client had a child whom she believed had died as an infant. Years later, an adult approached her claiming to be the child and seeking to join her in the United States. DNA testing was used to verify the relationship prior to submitting a petition for the child. While it alleviated the concerns of the attorney and client, the DNA test opened the client up to government suspicion. The question arose as to whether she had committed fraud in the past by not disclosing the existence of her child. DNA testing can help the government detect fraud, as in case 05. In this case, when the brother and sister samples submitted to a laboratory both profiled as male, and re-sampled as male, the unusual finding triggered an investigation; the investigation indicated that the same samples, known to be genetically related, were being intentionally submitted in place of actual samples of the family members. In case 06, the State Department reviewed submissions for different individuals from the same country and found that the same profile had been used for multiple cases.

Utility C: DNA testing requests or requirements can deter intentionally fraudulent misrepresentations of relationships or clarify misunderstandings around the kinds of relationships that qualify for immigration benefits

In case 7, IA18 claimed to have experienced cases where a request for DNA testing revealed that clients' social, legal, and genetic relationships did not align with how they had been presented in the petition. They described conversations with various clients, for instance, "Often it's, 'Well I raised, you know, Susan since she was an infant. Her mother is my sister and couldn't take care of … in fact, she's my niece.'" IA18 indicated that clients sometimes seemed to have intentionally concealed the true relationship and sometimes to have misunderstood the documentation and relationship required to support their petitions. IA12 described similar conversations, stating, "I certainly see those cases, in which case I've had to advise clients, you know, because that isn't your biological daughter…, and you aren't actually married to the mother, or just whatever circumstances, …you know, we're not able to move forward with that petition."

Pitfall A: Family is not defined by genetic relationships alone; kinship terms do not correspond to biological (or genetic) relationships in the same way across languages and cultures

NG21 captured this pitfall when describing the risks of DNA testing:

> Emotionally I think there are [risks]. I think—I'm not a scientist, but it seems like a pretty straightforward process as far as swabbing the inside of your cheek—I think what is maybe the problem is our immigration laws are written from a very Western, white perspective of what a family is.

A comment from NG02 further explained the appeal and risks of DNA evidence:

Table 4. Pitfalls of relationship and identity DNA testing across US immigration contexts

	Visa petition context	Unaccompanied youth context	Government separation context	Family verification context	Transnational missing context
No pitfalls stated or interpreted	cases 1–6	no case examples	no case examples	no case examples from interviews; case 25[a]	no case examples from interviews; case 26[a]
Pitfall A: family is not defined by genetic relationships alone; kinship terms do not correspond to biological (or genetic) relationships in the same way across languages and cultures	cases 7, 22[a]	no case examples	no case examples	no case examples	no case examples
Pitfall B: kinship analysis can reveal sensitive information	cases 8, 9, 10, 11, 12, 22[a]	no case examples	no case examples	no case examples	no case examples from interviews; case 27[a]
Pitfall C: collection, processing, and comparison of DNA samples from multiple individuals can carry logistical, temporal, geographical, and financial burdens	cases 13, 14, 15, 23,[a] 24[a]	no case examples	case 21	no case examples	no case examples from interviews; cases 27,[a] 28[a]
Pitfall D: DNA from the appropriate individuals to test a relationship is not always available	case 14	no case examples	no case examples	no case examples	no case examples from interviews; cases 28,[a] 29[a]
Pitfall E: the appropriate technology and/or infrastructure to test a relationship is not always available	cases 14, 15, 16	no case examples	no case examples	no case examples	no case examples from interviews; cases 28,[a] 29,[a] 30[a]
Pitfall F: the government might not collect samples or request or apply DNA testing results uniformly in decision-making processes	cases 12, 16, 17, 18, 19, 22,[a] 23,[a] 24[a]	case 20	no case examples	no case examples	no case examples from interviews; cases 27,[a] 29,[a] 30,[a] 31[a]

See Table 1 for context descriptions.
[a]Case examples sourced from the study team, not from interviews.

> You know, all this indica [*sic*] of, of parental relationship[s] and it's just, it's just easier for the government to have this clear objective evidence. Now I do admit that the government is always concerned about fraud…. But when they create a rule that they think is, well this is good, this, this will prove it, they're—they're ignoring the consequences of that rule—right, and the hardship. It could be economic hardship, it could be stress, it could be error in the testing. It could be that the child is not the biological child of the parent. So, um, I don't know why the law would privilege biology over behavior.

NG15 also noted that when genetic relationships do not match claimed relationships, petitioners might have their application denied or be labeled fraudulent. Two case examples demonstrate particularly well the extent family structures might not align with policy. IA18's conversations with petitioners in case 7, described above, indicated that there was confusion around relationship terms for some families. Case 22 highlighted a family with caregiving, genetic, and legal relationships that nevertheless faced barriers to immigration; a same-sex married couple, one a US citizen and one a foreign citizen, applied for US citizenship for their children born via surrogacy. Each child was genetically related to one parent; only the child genetically related to the US citizen was granted citizenship. This case was later reversed in courts, granting citizenship to both children.

Pitfall B: Kinship analysis can reveal sensitive information

DNA testing can reveal unknown or unexpected information about relationships (e.g., misattributed parentage). Participants also illustrated how conversations leading up to DNA testing could reveal sensitive information about family relationships. The effect of DNA test results on the immigration process, introduced under pitfall A above, and lack of preparation on the part of professionals to communicate results, might compound any trauma from the revelation of sensitive information. Case 9, in which a man wished to bring the child he had with a woman abroad to the United States, demonstrated all three of these aspects of this pitfall. A man revealed to his wife that he had a child abroad with another woman and filed a petition for the child. During the application process, DNA testing was requested and unexpectedly revealed that he was not the genetic father. IA01 described the father's reaction as "dumbfounded." With no genetic or legal connection to the child, the petition was denied; outside of undergoing a legal adoption process, the man was left with no options to bring the child to the United

States. IA01 summarized the effect of DNA testing on cases like this one:

> If the DNA turns out that, hey, that's not your child or that's not your parent or that's not your brother or sister, then, then that's really it. Then that person, regardless of what kind of emotional connection you have with that individual or however much time you spent, that person is just a random person in your life and they're not necessarily eligible for benefits.

Misattributed paternity also ended the petition under the Central American Migrant Minors Program of the father in case 10. NG21 alluded to the challenge of communicating results of nonpaternity to a man who believed he was the genetic father: "[The attorney] was one of my colleagues that I shared an office with. And, yeah, she was definitely not prepared to give him those results." Case 11 demonstrated another type of trauma that can emerge from the revelation of misattributed paternity. NG15 recalled three instances of misattributed paternity in a refugee resettlement center. These results not only ended the application process for the fathers in the cases but they often brought up trauma from rape among the women in the population served by the center.

The sensitive information that can be revealed by DNA testing includes more than misattributed parentage. In transnational missing persons cases, while finding a genetic relationship between an FRS and a UHR sample is a step toward identification, it also potentially confirms the death of a relative. The sensitivity of this information was particularly clear in case 27, where a family did not accept the DNA identification of a deceased migrant as their missing child. DNA identification is not unquestionable, nor is a relationship test. In fact, families should be permitted to question results of the tests. In case 27, the family's rejection of the identification could have stemmed from both emotion and their knowledge of their family member's case, or their reaction could reflect the sensitivity of communications in this context.

Not every case involving misattributed parentage results in a denial, but the revelation of sensitive information can still be traumatic even in an ultimately successful case. In case 12, a father with an established caregiving relationship with his child came forward as a sponsor when the child was held as an unaccompanied minor in Office of Refugee Resettlement (ORR) custody. A DNA test requested during the application process revealed that he was not the genetic father. This case example also captures the lack of preparedness to communicate unexpected results, as narrated by NG06:

> In that case, the government also agreed to leave the decision to disclose with the putative parent, or the caretaker parent, so that he could decide when and how to tell his daughter that there was no biological relationship, because I think they [the ORR] had considered telling her while she was detained and in custody without anyone to be around her to support her to, through learning that information. As if detention isn't difficult enough for a kid.

Ultimately, the existing caregiving relationship prompted ORR to release the child to him. The couple in case 22, described above, never intended to learn which child was related to which father. The decision by the State Department to grant citizenship to only one child forced them to reveal which child was genetically related to whom, information they had planned to never share, to the government and ultimately the public.

Pitfall C: Collection, processing, and comparison of DNA samples from multiple individuals can carry logistical, temporal, geographical, and financial burdens

In some of the cases described, DNA testing was burdensome not because of any inherent characteristic of the process but because of the circumstances in which families had to carry out testing. For instance, while IA13 described the current costs of DNA testing as "reasonable," five other participants (NG02, IA10, IA11, IA12, and NG15) all contextualized the financial burden of DNA testing within the means of families, particularly low-income and refugee families. They also described instances where DNA testing created burdens due to the structure and history of a family. IA12 noted that the financial burden of testing might be dependent on how many relationships a family needed to test. In case 14, IA23 hoped to compare a client's DNA data to several half-siblings, but a test had yet to be completed because the siblings were dispersed. Similarly, the adoptee in case 23 spent over a year tracking down his biological siblings in the hopes of using a DNA test as evidence of his US citizenship. Location of family members can also exacerbate challenges that make DNA testing burdensome. In case 13, a young child was left without a proper guardian upon the death of the mother; sample collection in this circumstance was challenging, as the father was in the United States and the child was in a refugee camp abroad. Failure of governments or other organizations to provide appropriate resources or infrastructure also creates burdens. In case 24, border closures and travel restrictions due to the coronavirus prevented a couple from promptly gathering the necessary evidence to finalize an adoption. In addition, the US Embassy in the country where one birth parent was located was reticent to facilitate DNA testing. In the transnational missing context, families might face open-ended waiting times for kinship associations even after samples have been collected and submitted. In case 27, UHR were first exhumed and sampled 7 years after burial; an association was first made about a year after DNA data from the UHR sample were uploaded to a database. In case 28, a DNA sample was collected by a medical examiner and genotyped for STRs, but no kinship matches have yet resulted.

Pitfall D: DNA from the appropriate individuals to test a relationship is not always available

This might be due to a death, as in case 14, or to other challenges to sample collection and comparison. IA23 cited a request for a specific antigen test of a decedent as "…an example of…how little prepared the court, to the—the USCIS officers are really. How little informed they are of what some of these tests mean." In the transnational missing context, FRSs and UHR samples must be entered into the same database for an association to be made. In cases 28 and 29, DNA samples were collected from UHR, but no kinship matches had resulted, potentially because the families had not provided samples, or they had provided samples to a database that was not compared to the one holding the DNA data from the remains.

Pitfall E: The appropriate technology and/or infrastructure to test a relationship is not always available

This might include stakeholders' understanding of testing methodologies, established procedures for requesting and submitting DNA test results, systems for locating and collecting samples, or data management challenges. IA23 described the reaction of USCIS in case 14: "So, we did the blood test with my client who was father/mother A with a half sibling father/mother B and another half sibling father/mother C to establish that, you know, he was, you know, was the father. And USCIS couldn't wrap its head around that." IA23 expressed surprise that "they had difficulty" with complex kinship methodologies for demonstrating paternity. NG06 recalled a series of difficult cases out of one East African country, captured in case 15. This country had no paperwork available for ordering a DNA test and no paperwork or system for locating relatives. In case 16, DNA testing was successfully carried out, but submission to USCIS posed a problem. The client and his attorney (IA17) submitted a DNA test before it was requested by USCIS, knowing they had no other documentation of a father-child relationship, but USCIS responded with a request for a birth certificate listing the names of both parents. There did not seem to be a structure in place to allow DNA test results to be submitted up front. TC08 and 09 noted, "Generally the hearsay that I have would be that this [process is] document-driven and then DNA is [requested] towards the end. Documents failed and…then they'll go to the DNA." For transnational missing persons, as in unsolved cases 28 and 29, even if FRSs and UHR samples were genotyped and databased, current database structures and policies created information silos that decreased the likelihood of successful associations being made.

Pitfall F: The government might not collect samples or request or apply DNA testing results uniformly in decision-making processes

Specific cases described by participants capture how inconsistencies in DNA testing and decision-making processes might manifest. Inconsistencies included instances where DNA tests were requested to evaluate explicitly non-genetic relationships, where the study team found contrasts between the use of DNA in similar cases or where participants felt DNA evidence was weighted in an unexpected or precedent-breaking way, and where policies or laws around the collection, processing, or evaluation of DNA evidence were not followed. Case 19 provides an example of an unreasonable request for DNA evidence, in which USCIS erroneously requested a DNA test of a husband-wife couple. Their attorney (NG21) brought the request to the attention of a USCIS officer, who attributed the request to clerical error. A series of cases where DNA evidence was used to determine with whom a child could reside demonstrates the shifting value placed on paper documentation, histories of caregiving relationships, and DNA evidence. In case 21, NG02 suggested that new DNA requirements put in place by HHS complicated the reunification of parents and children separated under the zero-tolerance immigration policy. While HHS required DNA testing for government-separated children in case 21, in case 12 in the case of an unaccompanied youth, a caregiving relationship was sufficient to release a child to a sponsor despite negative paternity results. In the visa petition context, in both cases 16 and 17, fathers with little or no contact with their children submitted petitions but had little access to paper documentation because they had fled wars. In case 16, results of paternity submitted ahead of a request were not considered sufficient evidence, and in case 17, NG15 was concerned that DNA testing would not help the petition since the father and child had long been estranged and there was little paper documentation available. IA13 highlighted a scenario (case 18) in which, despite DNA evidence of paternity, a father's petition for his child was rejected based on the law in the country of origin, which required demonstrated financial support and cohabitation. The decision was ultimately overturned in accordance with US law. In contrast, in case 23 a man remained in danger of being deported despite submitting DNA evidence of his biological relationship with six full siblings, all born to two US citizen parents, together with medical records, a birth certificate, documents related to his name change upon adoption, and statements from his adoptive and biological family members. Some case descriptions indicated that government or state officials themselves obstructed DNA collection. In case 24, described above, a US Embassy did not cooperate in the DNA collection required to finalize an intercountry adoption. In addition, in the transnational missing persons context, in cases 27, 29, 30, and 31, DNA samples were initially not collected from UHR despite state law requiring DNA sampling of unidentified corpses.

Participant reflections on the weight of DNA testing as evidence captured additional inconsistencies. Participants relayed contrasting assessments of how they might advise clients and how they thought the government weighted DNA evidence. These assessments were closely interwoven with the perceived reliability of evidence, particularly in connection with characteristics of families and their countries of origin. IA11 stated,

> It's [DNA evidence] heavily, heavily weighted. The government has never ever pushed back on a DNA test result to me. …They've pushed back on all kinds of other documents we've submitted, but not the DNA test, they have never pushed back about its credibility. …And then I've—I've always found it interesting; how do they know this company is reputable? Right.

IA12 stated that they always advised DNA testing if financially possible when paper documentation dating a child's birth was unavailable; in the absence of strong paper documentation, USCIS sees DNA as the "most conclusive evidence." IA19 reflected a more even weight of DNA evidence in comparison to other forms of documentation, indicating that "it weighs the same" and that "a birth certificate with the father's name on it that is, you know, authenticated and it's real is, is acceptable evidence. Again, that's not—you know, just like a DNA test, that's not 100 percent either, you know"? Similarly, IA01 described DNA testing as "one tool in an entire toolbox of ways in which to make a connection."

Differences in the weighting of DNA testing take on significance when consent and discrimination are considered. AC03 noted that while there are contexts where DNA testing was

> a reasonable and appropriate…measure…. Where we potentially get into trouble is in situations where the agency begins demanding DNA testing to verify family relationships, not because it thinks there might be a reason for doubt on [a] case-by-case basis, but just because it's decided than an entire class of cases, like the case—you know, refugees from South West Africa, say—[might be fraudulent].

The concept of "high-fraud countries" (IA04) ran throughout several interviews to varying degrees. IA11 drew a relationship between requests for DNA testing, other forms of documentation, the reputation of certain regions, and the possibility of discrimination. He related that "in the vast majority of cases that I've used DNA testing to prove biological relationships, they were—the people were of African descent…um, and Black African descent, not white South Africa or North African Arabs. They were Black Africans." When asked why that might be, IA11 responded, "There's a tremendous amount of doubt about the paperwork that comes from a lot of those countries in Africa. The general or common thought is that…there's a lot of fraudulent documentation being purchased and used." He concluded, "And so, those countries that are less developed, the record-keeping is less trusted, then the government will push back and you have to use DNA testing to prove the relationships." IA10 raised the issue of consent for the families that are presented with requests for DNA testing:

> I don't feel—I feel like if immigration requests it, I don't feel like you have an option to say no. Because if you say no, then they're going to infer that it's not a real relationship…. And so I don't think there are, you know, circumstances where I would tell a client not to do it. Because they've already assured me that this person is their child and everything, so you know, to some degree if they want the case approved, I mean they have to get—they have to do it. You know? And I am not sure that there's really a choice."

Discussion

Our findings provide insight into the distinct utility of genetic information in a non-medical context, as well as the potential pitfalls of using genetic information as a proxy for family relationships. They also illustrate the interplay of utilities and pitfalls in individual cases and how they affect petitioners and professional stakeholders. By identifying utilities and pitfalls in known cases and pinpointing those that could apply across contexts, we provide a framework to aid in the development of guidelines in emergent application contexts.

We were unable to access the full range of stakeholders who might be involved with DNA testing for immigration, particularly government officials or agents, for interviews. The challenges of identifying and contacting government officials aligns with the lack of transparency about the details of DNA testing processes in immigration contexts. Our information-oriented sampling yielded an insufficient number of immigration attorneys, which prompted our adjusted sampling approach. Most cold email invitations went unanswered, but some responded that they did not have experience with DNA testing, despite our instruction that experience was not a requisite for participation. This speaks to the lack of broad uptake of DNA tests in immigration practices and possibly to the ignorance of the availability of DNA tests as a source of evidence, although not all immigration law involves family petitions. The immigration attorneys also expressed the limits of their knowledge and experience of DNA testing despite working in the longest-standing DNA testing context. The combination of their emphasis of their lack of experience and assertions of the power of DNA evidence when used also indicates a need for tools for understanding the potential implications of the technology.

Our interviews also did not capture case examples for every context. Tapping into news sources enabled us to highlight the utilities and pitfalls relevant to some of those contexts, but a systematic media analysis or further interviews would likely expand the understanding of these contexts. Nevertheless, our framework is rooted in qualitative analysis of the experiences of the participants. Further interviews are also warranted by the ongoing rapid developments in this sphere.

The potential utility of a DNA test in helping a family support their immigration case, particularly when initiated

by the family (rather than required by authorities), might well balance the risk of some of the pitfalls arising, depending on the case. Inflexible definitions of family and lack of transparency around DNA testing applications in immigration, however, remain primary concerns. A broader definition of family[5] is needed in immigration policies to avoid some of the pitfalls outlined in our results. DNA testing should be used in tandem with, not in place of, other forms of documentary evidence and should not by itself be used to support exclusion from immigration benefits. A lack of transparency in how DNA tests are weighted in cases and what is termed fraud across immigration contexts hampers the development of guidelines that draw on input from the broad array of implicated stakeholders. Lack of transparency on how the government defines fraud in terms of family units compounds the pitfalls of applying DNA data to these cases.[8] In the rapid DNA testing for family relationship verification at border entry points, it remains unclear how families are selected for testing and evaluated for fraud.[27]

As Holland[5] asserts, inflexible definitions of family are particularly prone to disadvantage migrant families, who endure circumstances that disrupt and re-form relationships of all kinds. With greater understanding of the processes, migrant advocates can better guide families on when DNA tests are necessary, and inconsistencies in the weight of DNA test results on decisions might dissipate. The balance of benefits and risks here is akin to the balance considered in health-related uses of genetic information to guide consent and pre- and post-communication of results. Both health-related and long-standing immigration contexts can provide a wealth of understanding to inform guidelines that allow access to the utility of relationship DNA testing in immigration contexts while minimizing pitfalls.

Data and code availability

There are restrictions to the availability of the interview data due to participant confidentiality and risks to the people whose immigration cases and family dynamics were discussed. Data are protected with a Certificate of Confidentiality. Case example narratives and citations to sources for cases in the public domain are available in supplemental information. Authors may contact the study team for de-identified transcripts.

Supplemental information

Supplemental information can be found online at https://doi.org/10.1016/j.xhgg.2021.100060.

Acknowledgments

The authors appreciate the efforts of Dan Berger and Amy Mundorff for consultation on the pitfalls; Dan Berger, Eddie Canales, Kate Spradley, and Don White for providing case examples relevant to transnational missing cases; and Zecilly Guzman for reading case example narratives draft. Authors S.H.K. and B.A.B. initiated this project while at the Initiative for Science and Society at Duke University, and D.M. and J.K.W. conducted much of this work at Geisinger's Center for Translational Bioethics and Healthcare Policy. This study was supported by the National Human Genome Research Institute grant R01HG009923.

Declaration of interests

Jennifer K. Wagner is on the Editorial Board of *HGG Advances*. All other authors declare no competing interests.

Received: August 12, 2021
Accepted: September 20, 2021

Web resources

DNA Bridge, https://www.dnabridge.org/
Rev, https://www.rev.com
American Immigration Lawyers Association, https://www.aila.org

References

1. Katsanis, S.H. (2020). Tracing windblown seeds: genetic information as a biometric for tacking migrants. In s. In Silent Witness: Applying Forensic DNA Analysis in Criminal and Humanitarian Disaster, H. Erlich, E. Stover, and T. White, eds. Silent Witness: Applying Forensic DNA Analysis in Criminal and Humanitarian Disaster (Oxford University Press), pp. 208–237.
2. Sperry, B.P., Allyse, M., and Sharp, R.R. (2017). Genetic fingerprints and national security. Am. J. Bioeth. *17*, 1–3.
3. Dove, E.S. (2013). Back to blood: The sociopolitics and law of compulsory DNA testing of refugees. University of Massachusetts Law Review *8*, 1–42.
4. Heinemann, T., Naue, U., and Tapaninen, A.-M. (2013). Verifying the family? A comparison of DNA analysis for family reunification in three European countries (Austria, Finland and Germany). Eur. J. Migr. Law *15*, 183–202.
5. Holland, E. (2011). Moving the virtual border to the cellular level: Mandatory DNA testing and the U.S. refugee family reunification program. Calif. Law Rev. *99*, 1635–1682.
6. Jeffreys, A.J., Brookfield, J.F., and Semeonoff, R. (1985). Positive identification of an immigration test-case using human DNA fingerprints. Nature *317*, 818–819.
7. Cronin, M.D. (2000). Guidance on parentage testing for family-based immigrant visa petitions. Immigration and Naturalization Service, U.S. Department of Justice. https://www.aila.org/infonet/ins-on-parentage-testing.
8. Esbenshade, J. (2010). Special report: An assessment of DNA testing for African refugees (Immigration Policy Center, American Immigration Council). https://www.americanimmigrationcouncil.org/research/assessment-dna-testing-african-refugees.
9. Bruno, A. (2018). Refugee Admissions and Resettlement Policies. Congressional Research Service. https://sgp.fas.org/crs/misc/RL31269.pdf.
10. Trump, D. (2017). Protecting the nation from foreign terrorist entry into the United States. Executive Order 13780.
11. Department of State (2014). Notice of information collection under OMB emergency review: Affidavit of relationship (AOR) for minors from Honduras, El Salvador and Guatemala. Notice of request for emergency OMB approval and public comment, 79 Fed. Reg., 68343-68343, November 14, 2014.

12. U.S. Citizenship and Immigration Services (2017). In-country refugee/parole processing for minors in Honduras, El Salvador and Guatemala (U.S. Citizenship and Immigration Services). https://www.uscis.gov/CAM.
13. Department of Homeland Security (2017). Termination of the Central American Minors Parole Program. Notice, 82 Fed. Reg., 38926-38927, August 16, 2017.
14. American Association of Blood Banks (2019). Standards for relationship testing laboratories, 14th edition, effective January 1, 2020 (American Association of Blood Banks).
15. 6 USC §279(g)(2). (2002).
16. 8 USC § 1232(c)(2). (2008).
17. Homeland Security Act of 2002. Sec 462, Public Law 107-296, 6 USC §101. (2002).
18. Office of Refugee Resettlement (2021). Unaccompanied children frequently asked questions (Office of Refugee Resettlement). https://www.acf.hhs.gov/orr/unaccompanied-children-frequently-asked-questions.
19. Department of Health and Human Services (2021). Proposed information collection activity; sponsor review procedures for unaccompanied alien children. Request for public comment, 86 Fed. Reg., 308-310, January 5, 2021.
20. Madden, D., and Katsanis, S.H. (2021). Letter to the Editor-Context-specific considerations for development of guidelines for the implementation of rapid DNA. J. Forensic Sci. *66*, 793–796.
21. 310 F. Supp. 3d 1133. Ms. L. v. U.S. Immigration & Customs Enforcement (2018). 310 F. Supp. 3d 1133, Case No. 3:18cv428 DMS MDD, United States District Court, Southern District of California.
22. Hargan, E.D. (2018). Letter to Representative Elijah Cummings (U.S. Department of Health and Human Services).
23. Family Reunification Task Force (2021). Family Reunification Task Force: 120-day initial progress report (U.S. Department of Homeland Security). https://www.dhs.gov/publication/family-reunification-task-force-120-day-initial-progress-report.
24. Joint Status Report. Ms. L, et al. vs. U.S. Immigration and Customs Enforcement (2021). Case No. 3:18cv428 DMS MDD, United States District Court, Southern District of California.
25. Barnert, E., Katsanis, S.H., Mishori, R., Wagner, J.K., Selden, R.F., Madden, D., Berger, D., Erlich, H., Hampton, K., Kleiser, A., et al. (2021). Using DNA to reunify separated migrant families. Science *372*, 1154–1156.
26. Department of Homeland Security (2019). Privacy Impact Assessment for the rapid DNA operational use. DHS/ICE/PIA-050, June 25, 2019. https://www.dhs.gov/publication/dhsicepia-050-rapid-dna-operational-use.
27. U.S. Department of Homeland Security (2020). Collection and use of biometrics by U.S. Citizenship and Immigration Services. Proposed Rule, 85 Fed. Reg, 56338-56422, September 11, 2020.
28. Aleaziz, H. (2021). Biden is going to scrap a Trump-era plan to force immigrants to submit eye scans, voice prints, and DNA. BuzzFeedNews. https://www.buzzfeednews.com/article/hamedaleaziz/biden-scrapping-trump-immigration-biometrics-plan.
29. Katsanis, S.H., and Spradley, M.K. (2020). Preventing a third death: Identification of missing migrants at the U.S.-Mexico border. In Silent Witness: Applying Forensic DNA Analysis in Criminal and Humanitarian Disasters, H. Erlich, E. Stover, and T. White, eds. (Oxford University Press), pp. 238–267.
30. Spradley, M.K., and Gocha, T.P. (2020). Migrant deaths along the Texas/Mexico border. In Forensic Science and Humanitarian Action: Interacting with the Dead and the Living, R.C. Parra, S.C. Zapico, and D.H. Ubelaker, eds. (Wiley Online Library), pp. 535–548.
31. Katsanis, S.H., Madden, D., Siegert, C.B., Canales, E., and Spradley, M.K. (2021). Building a policy scaffold for transnational missing DNA data sharing challenges. Forced Migr. Rev. *66*, 61–63.
32. U.S. Department of Justice, Office of Public Affairs (2018). Attorney General announces zero-tolerance policy for criminal illegal entry. Justice News. https://www.justice.gov/opa/pr/attorney-general-announces-zero-tolerance-policy-criminal-illegal-entry.
33. Sessions, J. (2018). Attorney General Sessions addresses recent criticisms of zero tolerance by church leaders. Justice News. https://www.justice.gov/opa/speech/attorney-general-sessions-addresses-recent-criticisms-zero-tolerance-church-leaders.
34. Flores, A. (2018). The US will resort to DNA tests in an effort to reunite children and their parents. BuzzFeed News. https://www.buzzfeednews.com/article/adolfoflores/the-feds-say-theyll-use-dna-testing-to-reunite-kids-and.
35. Wagner, J.K., Madden, D., Oray, V., and Katsanis, S.H. (2019). Conversations surrounding the use of DNA tests in the family reunification of migrants separated at the United States-Mexico border in 2018. Front. Genet. *10*, 1232.
36. Berger, D., Hu, M., Katsanis, S.H., and Wagner, J.K. (2021). Midnight regulations and mass biometric data collection by DHS. The Regulatory Review. https://www.theregreview.org/2021/03/11/berger-hu-katsanis-wagner-biometric-data-midnight-regulations/.
37. U.S. Customs and Border Protection (2021). U.S. Border Patrol Southwest Border Apprehensions by Sector. Newsroom. https://www.cbp.gov/newsroom/stats/southwest-land-border-encounters/usbp-sw-border-apprehensions.
38. Blackburn, M., and Gooden, L. (2021). End Child Trafficking Now Act. S. 903/H.R.2219.
39. Burke, W., Laberge, A.-M., and Press, N. (2010). Debating clinical utility. Public Health Genomics *13*, 215–223.
40. Katsanis, S.H., and Katsanis, N. (2013). Molecular genetic testing and the future of clinical genomics. Nat. Rev. Genet. *14*, 415–426.
41. Foster, M.W., Mulvihill, J.J., and Sharp, R.R. (2009). Evaluating the utility of personal genomic information. Genet. Med *11*, 570–574.
42. Department of Justice (2020). DNA sample collection from immigration detainees. Final Rule, 85 Fed. Reg., 13483-13493, March 9, 2020.
43. Oray, V., and Katsanis, S.H. (2021). Ethical considerations for DNA testing as a proxy for nationality. Glob. Bioet. *32*, 51–66.

HGGA, Volume *3*

Supplemental information

Framing the utility and potential pitfalls of relationship and identity DNA testing across United States immigration contexts

Diana Madden, Brianna A. Baker, Jennifer K. Wagner, and Sara H. Katsanis

Exhibit EI-G - Expert interview guide (*April 2018 protocol*)

INTERVIEWER: ________________________ STUDY ID: ________________________

DATE: ______________________

Thank you for agreeing to talk with us. As stated in the [email / letter / phone call] you received from us, we are collecting feedback from expert stakeholders who work with migrant families that may be providing DNA samples or undergo DNA tests. Our research team is conducting interviews with a number of people and we are interested in your opinions and experiences.

[REVIEW CONSENT FORM AS PER PROTOCOL]

1. To get things started, can you tell us what you know about how DNA tests are used in immigration?

2. What is your involvement with migrants or migrant families?

3. Do you order or help arrange DNA [tests / sample collection] for migrants or families?

4. [IF Q2 YES] What kind of DNA tests do you order? Who performs the tests? Who pays for tests?

5. Briefly, what is your understanding of what a DNA test will tell you?

6. How do you expect DNA test results to be helpful?

7. In what circumstances do you not suggest DNA tests?

8. Do you receive DNA test results?

9. [IF Q7 YES] With whom might you share the DNA test results?

10. Do you think there are any risks in taking a DNA test?

11. Likert Scale: On a scale of 1 to 5, how important DNA testing in immigration? With “1” being not important at all and “5” being very important

That concludes our session. Thank you so much for sharing your thoughts and opinions with us. If you have additional information that you did not get to say today, please contact one of us.

CASE EXAMPLE CATALOG

PARTICIPANT-SOURCED CASE EXAMPLES **2**
CONTEXT 1: DNA TESTING FOR RELATIONSHIP OR IDENTITY VERIFICATION FOR VISA OR CITIZENSHIP APPLICATIONS AND PETITIONS FOR NONCITIZEN RELATIVES 3
Case 1: DNA testing documents a relationship *3*
Case 2: DNA testing documents a relationship *3*
Case 3: DNA testing documents a relationship *4*
Case 4: DNA testing disproves fraud *4*
Case 5: DNA testing detects fraud *5*
Case 6: DNA testing detects fraud *5*
Case 7: DNA testing requests deter fraud *5*
Case 8: DNA testing disproves fraud and reveals sensitive information *6*
Case 9: DNA testing reveals misattributed paternity *6*
Case 10: DNA testing reveals misattributed paternity *7*
Case 11: DNA testing reveals misattributed paternity *7*
Case 12: DNA testing reveals misattributed paternity *8*
Case 13: DNA testing is burdensome, but documents a relationship *8*
Case 14: DNA testing is burdensome, but could document a relationship *9*
Case 15: DNA testing is burdensome *9*
Case 16: DNA documentation of a relationship is insufficient *10*
Case 17: DNA documentation of a relationship could be insufficient *11*
Case 18: DNA documentation of a relationship is insufficient *11*
Case 19: DNA testing is requested erroneously *12*
CONTEXT 2: DNA TESTING FOR RELATIONSHIP VERIFICATION FOR PLACEMENT WITH SPONSORS OF UNACCOMPANIED MIGRANT MINORS 13
Case 20: DNA testing is not requested uniformly across similar cases *13*
CONTEXT 3: DNA TESTING FOR VERIFICATION OF PARENT-CHILD RELATIONSHIPS FOLLOWING GOVERNMENT-IMPOSED FAMILY SEPARATION 14
Case 21: DNA testing is burdensome *14*
STUDY TEAM-SOURCED CASE NARRATIVE EXAMPLES **15**
CONTEXT 1: DNA TESTING FOR RELATIONSHIP OR IDENTITY VERIFICATION FOR VISA OR CITIZENSHIP APPLICATIONS AND PETITIONS FOR NONCITIZEN RELATIVES 16
Case 22: DNA testing is requested erroneously *16*
Case 23: DNA documentation of a relationship is insufficient *17*
Case 24: Officials will not collect a DNA sample *18*
CONTEXT 4: DNA TESTING FOR VERIFICATION OF PARENT-CHILD RELATIONSHIPS AT BORDER ENTRY POINTS 19
Case 25: DNA testing request reveals fraud *19*
CONTEXT 5: COMPARISON OF FAMILY REFERENCE SAMPLES TO UNIDENTIFIED HUMAN REMAINS SAMPLES FOR IDENTIFICATION PURPOSES IN TRANSNATIONAL MISSING PERSONS CASES 20
Case 26: DNA testing documents a genetic relationship *20*
Case 27: DNA testing documents a relationship, but is rejected by the family *20*
Case 28: Infrastructure delays or prevents a DNA identification *21*
Case 29: Infrastructure delays or prevents a DNA identification *22*
Case 30: Infrastructure delays or prevents a DNA identification *23*
Case 31: Officials will not collect a DNA sample *23*

This report was prepared as part of the Genomics, Biometrics, and Identity project, at Lurie Children's Hospital and Northwestern University. Team members include SH Katsanis, D Madden, JK Wagner, D Berger, MK Spradley, E Canales, B Baker, A Porter, and Z Guzman. This project was funded by the National Human Genome Research Institute R01HG009923.

PARTICIPANT-SOURCED CASE EXAMPLES

The following case example narratives highlight the utilities and pitfalls of DNA testing for relationships and identity in three of five U.S. immigration contexts where it is applied. These narratives were developed from descriptions of specific cases in interviews with professional stakeholders in immigration in the United States conducted in 2019. Each case narrative has been coded with the applicable overarching utilities and pitfalls of DNA testing. Codes were assigned from the perspective of the participant, based on participant statements about the specific utility and pitfalls of DNA testing in the case.

All case examples have been anonymized. Names are not included and are replaced with relevant kinship terms or a randomly assigned letter where necessary. Dates and gender are only included where judged necessary to support the narrative. Where a country of origin outside of the United States was specified, we include the region of the world where the country is located. All case examples occurred in the context of the U.S. immigration system.

Participant-sourced case examples are presented in the following format:

Assigned case number: Descriptive caption

**Source**	Code assigned to participant (participant's profession and assigned number)
**Region**	Region of the world where country or countries involved in the case are located (excluding the United States)
**DNA test type**	Retrospective/prospective DNA test and laboratory type OR DNA test and laboratory type that would have been used had testing been carried out
**Relationship tested**	Familial relationship(s) (to be) tested
**Stated value**	Utility of DNA testing in the case based on participant statements
**Stated pitfall(s)**	Pitfall(s) of DNA testing in the case based on participant statements
**Narrative**	Anonymized narrative of the case developed from interview transcripts and follow-up with participants where required

CONTEXT 1: DNA testing for relationship or identity verification for visa or citizenship applications and petitions for noncitizen relatives

Case 1: DNA testing documents a relationship

**Source**	Interview with Immigration Attorney 19 [IA19]
**Region**	Unspecified
**DNA test type**	Relationship STR at a commercial AABB laboratory
**Relationship tested**	Father-child
**Stated value**	DNA testing can provide documentation of genetic relationships and identity when other forms of documentation are unavailable, inaccurate, or insufficient to meet the burden of evidence.
**Stated pitfall(s)**	No pitfalls stated
**Narrative**	A U.S. citizen child wished to petition for a non-U.S. citizen father to obtain a green card for the parent. The father was not listed on the child's birth certificate, however, so a DNA test was used to establish the biological relationship.

Case 2: DNA testing documents a relationship

**Source**	Interview with Immigration Attorney 04 [IA04]
**Region**	North America
**DNA test type**	Relationship STR at a commercial AABB laboratory
**Relationship tested**	Mother-child
**Stated value**	DNA testing can provide documentation of genetic relationships and identity when other forms of documentation are unavailable, inaccurate, or insufficient to meet the burden of evidence.
**Stated pitfall(s)**	No pitfalls stated
**Narrative**	A woman, hereafter referred to as A, had a child out of wedlock in her home country while still a teenager. Because A was unmarried, her parents insisted that they be listed as the parents on their grandchild's birth certificate. Both A and her child had the exact same family name: on paper, they appeared to be full siblings with the same family name and a large age gap between them. Both A and her child became eligible for a special visa for victims of crime, which could ultimately lead to a green card. Because A's child was still a minor, her attorney filed on behalf of A; however, for both A and her child to get the visa, it had to be established that they had a mother-child (not sibling) relationship. DNA testing confirmed that their relationship was mother and child.

Case 3: DNA testing documents a relationship

**Source**	Interview with NGO Representative 21 [NG21]
**Region**	North Africa and Central Africa
**DNA test type**	Relationship STR at a commercial AABB laboratory
**Relationship tested**	Mother-child
**Stated value**	DNA testing can provide documentation of genetic relationships and identity when other forms of documentation are unavailable, inaccurate, or insufficient to meet the burden of evidence.
**Stated pitfall(s)**	No pitfalls stated
**Narrative**	A husband and wife were processed for refugee status in the United States while in a North African country; the husband was named the principal applicant. Their marriage was found valid, and they entered the United States together. Once in the United States, they decided to petition for the wife's biological children (the husband's stepchildren), whom they had left behind in a third country in Central Africa, to join them. The husband, having been named the principal applicant for refugee status, had to be the one to file the petition even though the children were not biologically his. The wife had to consent to a DNA test to show that the children were biologically hers; because the marriage had already been deemed valid, the DNA test confirmed that the petitioner was the stepfather.

Case 4: DNA testing disproves fraud

**Source**	Interview with Immigration Attorney 11 [IA11]
**Region**	West Africa
**DNA test type**	Relationship STR at a commercial AABB laboratory
**Relationship tested**	Unclear
**Stated value**	DNA testing can disprove or detect fraud when claimed relationships or identity are in question.
**Stated pitfall(s)**	No pitfalls stated
**Narrative**	An attorney had a U.S. citizen client who had gone through the process of naturalization in the United States. U.S. Citizenship and Immigration Services [USCIS] claimed that the client was not who they said they were and had not been eligible for the lawful permanent residence or green card status that gave rise to naturalization. The government thus wanted to denaturalize the person and rescind their lawful permanent resident status. The attorney had the client use a DNA test to verify their identity, thereby demonstrating that they were eligible for the status that originally led to naturalization.

Case 5: DNA testing detects fraud

**Source**	Interview with Technology Company Representatives 07 and 08 [TC08 and TC09]
**Region**	South Asia
**DNA test type**	Relationship STR at a commercial AABB laboratory
**Relationship tested**	Brother-sister
**Stated value**	DNA testing can disprove or detect fraud when claimed relationships or identity are in question.
**Stated pitfall(s)**	No pitfalls stated
**Narrative**	The DNA of a man and woman claiming to be brother and sister was tested, and the profiles derived from the samples were both male. Since the second sample should have been female, the laboratory requested a resampling, and the results profiled the same way. It was suspected that somebody was substituting the samples to cover up the unrelatedness of the two supposed siblings.

Case 6: DNA testing detects fraud

**Source**	Interview with Technology Company Representative 07 [TC07]
**Region**	Unspecified
**DNA test type**	Relationship STR at a commercial AABB laboratory
**Relationship tested**	Parent-child
**Stated value**	DNA testing can disprove or detect fraud when claimed relationships or identity are in question.
**Stated pitfall(s)**	No pitfalls stated
**Narrative**	A third party in an unspecified country was submitting the same known related samples to different laboratories for multiple cases. The State Department reviewed the results from several different individuals from that same country and realized that the same profile had been used repeatedly.

Case 7: DNA testing requests deter fraud

**Source**	Interview with Immigration Attorney 18 [IA18]
**Region**	Unspecified
**DNA test type**	Relationship STR at a commercial AABB laboratory
**Relationship tested**	Avuncular
**Stated value**	DNA testing requests or requirements can deter intentionally fraudulent misrepresentations of relationships or clarify misunderstandings around the kinds of relationships that qualify for immigration benefits.

**Stated pitfall(s)**	Family is not defined by genetic relationships alone; kinship terms do not correspond to biological (or genetic) relationships in the same way across languages and cultures.
**Narrative**	An attorney has had cases where clients revealed after receiving a government request for a DNA test that they were petitioning for biological nieces or nephews, not biological children. The attorney stated that sometimes it seemed to be intentional concealment of the true relationship by the petitioner and sometimes misunderstanding of the documentation (and relationship) needed to support their petitions. The attorney recommends to clients who are not the biological parents of a child to withdraw their applications rather than comply with requests for DNA testing that could reveal non-parentage and thereby risk being accused of fraud.

Case 8: DNA testing disproves fraud and reveals sensitive information

**Source**	Interview with Immigration Attorney 01 [IA01]
**Region**	South Asia
**DNA test type**	Relationship STR at a commercial AABB laboratory
**Relationship tested**	Mother-child
**Stated value**	DNA testing can disprove or detect fraud when claimed relationships or identity are in question.
**Stated pitfall(s)**	Kinship analysis can reveal sensitive information.
**Narrative**	In South Asia, the husband of a woman died while their baby was an infant. The husband's family took the child and told the woman that the baby also had died. Not realizing her child was still alive, the woman eventually immigrated to the United States, where she remarried. Decades later, she was contacted by an individual from South Asia who thought they were her child; the individual said they had been looking for her for the past 15 years. Because of the unusual circumstances, her attorney recommended a DNA test to make sure the claim was not a scam. The person turned out to be her biological child, and she filed a petition on their behalf. The question emerged as to whether the woman had committed fraud in the past by not disclosing the existence of the child, whom she had presumed dead.

Case 9: DNA testing reveals misattributed paternity

**Source**	Interview with Immigration Attorney 01 [IA01]
**Region**	West Africa
**DNA test type**	Relationship STR at a commercial AABB laboratory
**Relationship tested**	Father-child
**Stated value**	No utility stated
**Stated pitfall(s)**	Kinship analysis can reveal sensitive information.

**Narrative**	A married man living in the United States believed he had fathered child abroad out of wedlock. He revealed the existence of the child to his wife and decided he wanted to bring the child to the United States. He opened a petition, and a DNA test was requested. The results unexpectedly revealed that the child was not his biological child. Because he had no legal connection to the child (he was not married to the child's mother) and ultimately no biological connection, the petition was denied. His immigration attorney suggested that he could go through a separate process to adopt the child to establish a legal relationship.

Case 10: DNA testing reveals misattributed paternity

**Source**	Interview with NGO Representative 21 [NG21]
**Region**	Central America
**DNA test type**	Relationship STR at a commercial AABB laboratory
**Relationship tested**	Father-child
**Stated value**	No utility stated
**Stated pitfall(s)**	Kinship analysis can reveal sensitive information.
**Narrative**	A father applied to bring his child to the United States through the Central American Migrant Minors [CAM] program. The mother was in the country of origin, and the father was in the United States. The father, realizing he had the opportunity to bring his child to a safe place through the CAM program, petitioned for the child. DNA testing was required and revealed he was not the biological father; he thus had no recourse to get the child to the United States. His immigration attorney reportedly was not prepared to give him results of non-paternity.

Case 11: DNA testing reveals misattributed paternity

**Source**	Interview with NGO Representative 15 [NG15]
**Region**	Unspecified
**DNA test type**	Relationship STR at a commercial AABB laboratory
**Relationship tested**	Father-child
**Stated value**	No utility stated
**Stated pitfall(s)**	Kinship analysis can reveal sensitive information.
**Narrative**	A coordinator in a refugee resettlement center recalled at least three instances of misattributed paternity, which in the population they worked with was generally connected with rape. They described three cases in which fathers believed that children were biologically theirs, but DNA testing revealed misattributed paternity. Reporting misattributed paternity would often cause women to revisit rape trauma in the population the center served. In these cases, the

	coordinators would sit down and talk about the DNA test results with the clients. Misattributed paternity put an end to the immigration application process for the fathers.

Case 12: DNA testing reveals misattributed paternity

**Source**	Interview with NGO Representative 06 [NG06]
**Region**	Unspecified
**DNA test type**	Relationship STR at a commercial AABB laboratory
**Relationship tested**	Father-child
**Stated value**	No utility stated
**Stated pitfall(s)**	Kinship analysis can reveal sensitive information. The government might not request or apply DNA testing results uniformly in decision-making processes.
**Narrative**	While a DNA test that verifies a claimed biological relationship between a child and someone stepping forward to claim that child from ORR weighs heavily with the government, one participant described a case where a care-giving relationship was sufficient to convince ORR to release a child to a sponsor. A father and child had a 15-year relationship and believed themselves to be biologically related, although the father was not entirely certain he was the biological father. DNA testing ultimately showed that they were not genetically related, but the fact that they had a care-giving father-child relationship, if not a biological one, and that he was the safest and most appropriate placement for the child convinced the government to release the child to him. In this case, the government left the decision to disclose the results of the DNA test to the child up to the father, although the government also initially considered informing the child while they were in detention.

Case 13: DNA testing is burdensome, but documents a relationship

**Source**	Interview with Immigration Attorney 17 [IA17]
**Region**	East Africa
**DNA test type**	Relationship STR at a commercial AABB laboratory
**Relationship tested**	Father-child
**Stated value**	DNA testing can provide documentation of genetic relationships and identity when other forms of documentation are unavailable, inaccurate, or insufficient to meet the burden of evidence.
**Stated pitfall(s)**	Collection, processing, and comparison of DNA samples from multiple individuals can carry logistical, temporal, geographical, and financial burdens.
**Narrative**	A man came to the United States and petitioned for his wife and child to join him. The wife and child were in a refugee camp abroad; the

	wife died of malaria in the camp, leaving the child in the care of a very young relative and without a proper guardian. The birth certificate issued by the refugee camp was not considered sufficient evidence of the relationship between the father and child by USCIS; USCIS requested a birth certificate from the country the family fled as well as DNA evidence. Coordinating the logistics of obtaining DNA from the child in the refugee camp was extremely difficult given the circumstances. The petition was ultimately approved when DNA testing verified the father's relationship with the child.

Case 14: DNA testing is burdensome, but could document a relationship

**Source**	Interview with Immigration Attorney 23 [IA23]
**Region**	North America
**DNA test type**	Relationship STR at a commercial AABB laboratory
**Relationship tested**	Father-child; half siblings
**Stated value**	DNA testing can provide documentation of genetic relationships and identity when other forms of documentation are unavailable, inaccurate, or insufficient to meet the burden of evidence.
**Stated pitfall(s)**	Collection, processing, and comparison of DNA samples from multiple individuals can carry logistical, temporal, geographical, and financial burdens. DNA from the appropriate individuals to test a relationship is not always available. The appropriate technology and/or infrastructure to test a relationship is not always available.
**Narrative**	A client claiming to be a U.S. citizen had a U.S. citizen father who had died, leaving behind the client and several alleged full and/or half-siblings. The client was deported several times by U.S. authorities despite having a birth certificate with the alleged father named. The identity of the client's mother was disputed as one of two sisters, but the alleged mothers also were deceased. Since the client's parents were all deceased, the attorney suggested a DNA test of the half-siblings related through the father along with the client to demonstrate the paternal relationship. However, since the client had been repeatedly deported and the siblings were dispersed, coordinating the DNA testing of the various parties was difficult to accomplish, and testing had yet to be completed. It was unclear whether DNA evidence from multiple siblings would be successful in demonstrating the client's paternal relationship and claim to citizenship.

Case 15: DNA testing is burdensome

**Source**	Interview with NGO Representative 06 [NG06]
**Region**	East Africa
**DNA test type**	Relationship STR at a commercial AABB laboratory

**Relationship tested**	Unspecified
**Stated value**	No utility stated
**Stated pitfall(s)**	Collection, processing, and comparison of DNA samples from multiple individuals can carry logistical, temporal, geographical, and financial burdens. The appropriate technology and/or infrastructure to test a relationship is not always available.
**Narrative**	An NGO worker described a series of difficult cases out of a country in East Africa. The worker indicated that there was no paperwork available for processing DNA testing and finding relatives in the country, but even more challenging, there was no system for finding family members to conduct DNA tests. They indicated that very often relationships were established through means other than DNA testing, such as interviews.

Case 16: DNA documentation of a relationship is insufficient

**Source**	Interview with Immigration Attorney 17 [IA17]
**Region**	Unspecified
**DNA test type**	Relationship STR at a commercial AABB laboratory
**Relationship tested**	Father-child
**Stated value**	DNA testing can provide documentation of genetic relationships and identity when other forms of documentation are unavailable, inaccurate, or insufficient to meet the burden of evidence.
**Stated pitfall(s)**	The appropriate technology and/or infrastructure to test a relationship is not always available. The government might not collect samples or request or apply DNA testing results uniformly in decision-making processes.
**Narrative**	A father, now a naturalized U.S. citizen, fled civil war in his home country, leaving behind his child. Because he was forced to flee, he was not present at the birth of the child and was not listed on the child's birth certificate. He had, however, maintained contact with the child. After the child had grown a bit older, the father decided to petition for the child to join him. The father and attorney decided to submit a DNA test upfront, knowing that they lacked other forms of documentation of the relationship and knowing that the father had the means to pay for a DNA test. A DNA test was done, confirmed the claimed relationship, and was submitted to USCIS. USCIS sent a request for further documentation even though they already had the DNA test as proof of the relationship; specifically, they asked for a birth certificate with the names of both parents.

Case 17: DNA documentation of a relationship could be insufficient

**Source**	Interview with NGO Representative 15 [NG15]
**Region**	East Africa
**DNA test type**	Relationship STR at a commercial AABB laboratory
**Relationship tested**	Father-child
**Stated value**	DNA testing can provide documentation of genetic relationships and identity when other forms of documentation are unavailable, inaccurate, or insufficient to meet the burden of evidence.
**Stated pitfall(s)**	The government might not collect samples or request or apply DNA testing results uniformly in decision-making processes.
**Narrative**	Due to war, a father from East Africa living in the United States had been estranged from his child since the child was six months old. The child's mother was dead. Due to the estrangement, there was little documentation outside of a DNA test to establish the claimed relationship. The attorney representing the child questioned whether a DNA test would be relevant or useful in establishing with whom the child could reside, since the child does not know the father.

Case 18: DNA documentation of a relationship is insufficient

**Source**	Interview with Immigration Attorney 13 [IA13]
**Region**	Caribbean
**DNA test type**	Relationship STR at a commercial AABB laboratory
**Relationship tested**	Father-child
**Stated value**	DNA testing can provide documentation of genetic relationships and identity when other forms of documentation are unavailable, inaccurate, or insufficient to meet the burden of evidence.
**Stated pitfall(s)**	The government might not collect samples or request or apply DNA testing results uniformly in decision-making processes.
**Narrative**	A man from a Caribbean country living in the United States petitioned for his child (a minor) to join him; he did all the paperwork himself as he could not afford counsel. He was an American citizen, but his name did not appear on the child's birth certificate. A request for DNA testing was issued, authorized, and properly carried out by USCIS. DNA testing confirmed the father and child's genetic relationship. Despite the DNA match, the petition was denied by the United States based on the law in the country of origin, which required demonstrated financial support of the child and a demonstrated period of cohabitation. Upon appeal, the decision was overturned in favor of uniform application of U.S. law to petitions without regard to legal qualifications for parenthood in the country of origin.

Case 19: DNA testing is requested erroneously

**Source**	Interview with NGO Representative 21 [NG21]
**Region**	East Africa
**DNA test type**	Relationship STR at a commercial AABB laboratory
**Relationship tested**	Husband-wife
**Stated value**	No utility stated
**Stated pitfall(s)**	The government might not collect samples or request or apply DNA testing results uniformly in decision making processes.
**Narrative**	A husband and wife applied to come to the United States and received a request for DNA evidence from a USCIS service center. Their attorney found it odd, but they ultimately complied. When the attorney brought the request to the attention of a USCIS officer, the officer agreed that it was inappropriate and said that they would do retraining to avoid such requests in the future. The attorney initially thought that the request might be due to suspicion of fraud on USCIS's part, especially since the couple's country of origin only began producing civil issue documents accepted by the U.S. government in the mid-2010s; it was conceivable that a brother-sister pair without documentation of their relationship might try to apply as husband and wife. Upon further discussion with a more senior USCIS official, however, the attorney began to suspect that this was not an instance where USCIS suspected fraud, but rather that USCIS personnel had simply copy-pasted evidence requests without regard to the details of the case.

CONTEXT 2: DNA testing for relationship verification for placement with sponsors of unaccompanied migrant minors

Case 20: DNA testing is not requested uniformly across similar cases

**Source**	Interview with Immigration Attorney 16 [IA16]
**Region**	Unspecified
**DNA test type**	Relationship STR at a commercial AABB laboratory
**Relationship tested**	Mother-child
**Stated value**	No utility stated
**Stated pitfall(s)**	The government might not collect samples or request or apply DNA testing results uniformly in decision-making processes.
**Narrative**	An unaccompanied minor presented at the border hoping to go to the mother, located in the United States. The mother was asked to provide DNA to prove the relationship before picking up the child up from the Office of Refugee Resettlement [ORR] housing facility. The attorney stated that in other similar cases, DNA testing was not required for release to a sponsor and was not sure why it was part of the process in this case. The child was held by the U.S. Department of Health and Human Services [HHS] until the relationship was confirmed.

CONTEXT 3: DNA testing for verification of parent-child relationships following government-imposed family separation

Case 21: DNA testing is burdensome

**Source**	Interview with NGO Representative 02 [NG02]
**Region**	Central America
**DNA test type**	Relationship STR at a commercial AABB laboratory
**Relationship tested**	Parent-child
**Stated value**	No utility stated
**Stated pitfall(s)**	Collection, processing, and comparison of DNA samples from multiple individuals can carry logistical, temporal, geographical, and financial burdens.
**Narrative**	After the 2018 separations of children from family members traveling from Central America, one NGO reportedly agreed to take on 63 cases of separated children in attempts to reunify them. ORR used to consider a birth certificate or affirmation enough to release a child from their custody, but HHS had added a DNA requirement that created difficulties for the NGO in reuniting children with parents. Of the 63 family separation cases, only one or two involved relationships other than parent-child. In 15 of the 63 cases, a parent located in the United States wished to claim the child, and DNA testing was required to verify the claimed relationships. The NGO worked to discuss the process with children who were of the age of consent and able to communicate as well as to locate parents in detention. While the majority were reunited with their parents, a handful of them were unable to be reunited and required separate legal remedies to be released, either to a more distant relative, like an aunt or an uncle, or to another parent that was in the United States.

STUDY TEAM-SOURCED CASE NARRATIVE EXAMPLES

The following supplemental case example narratives highlight the utility and pitfalls of DNA testing in three of five U.S. immigration contexts where it is applied. These narratives were developed from cases provided by the study team. Each case narrative has been coded with the applicable overarching utilities and pitfalls of DNA testing. Utilities and pitfalls were interpreted by the study team based on the available information.

Many of these cases are publicly available in academic, media, or government reports; where cases are publicly available, a citation is provided, but the catalogue contents are anonymized as in the participant-sourced case examples. Cases not publicly available are cases in which a member of the study team is directly involved. In all cases, names are not included and are replaced with relevant kinship terms or a randomly assigned letter where necessary. Dates and gender are only included where judged necessary to support the narrative. Where a country of origin outside of the United States was specified, we include the region of the world where the country is located. All case examples occurred in the context of the U.S. immigration system.

Study-team sourced case examples are presented in the following format:

Assigned case number: Descriptive caption

**Source**	Study team reported or communication
**Region**	Region of the world where country or countries involved in the case are located (excluding the United States)
**DNA test type**	Retrospective/prospective DNA test and laboratory type OR DNA test and laboratory type that would have been used had testing been carried out
**Relationship tested**	Familial relationship(s) (to be) tested
**Stated value**	Utility of DNA testing in the case as interpreted by the study team
**Stated pitfall(s)**	Pitfall(s) of DNA testing in the case as interpreted by the study team
**Narrative**	Anonymized narrative of the case developed from details provided by the study team and/or publicly available information. Relevant citations to media, academic, or government reports provided.

CONTEXT 1: DNA testing for relationship or identity verification for visa or citizenship applications and petitions for noncitizen relatives

Case 22: DNA testing is requested erroneously

**Source**	Study team reported
**Region**	North America and Middle East
**DNA test type**	Relationship STR at a commercial AABB laboratory
**Relationship tested**	Father-child
**Interpreted value**	No utility interpreted
**Interpreted pitfall(s)**	Family is not defined by genetic relationships alone; kinship terms do not correspond to biological (or genetic) relationships in the same way across languages and cultures. Kinship analysis can reveal sensitive information. The government might not collect samples or request or apply DNA testing results uniformly in decision-making processes.
**Narrative**[1]	Twins Child 1 and Child 2 were born abroad to a married couple, Father 1 and Father 2, via surrogate using an anonymous donor's eggs and sperm from each father. Father 1 is a U.S. citizen, and Father 2 is a citizen of a Middle Eastern country. When the couple applied for U.S. passports for their newborn children in preparation to return to the U.S., DNA testing was requested to demonstrate both children's connection to Father 1 even though the couple had already been asked to explain how the twins were conceived and were legally married. The couple had planned never to reveal which child was genetically related to whom, even to the children themselves, but carried out the DNA testing hoping to obtain passports for their children. The results demonstrated that Child 1 was genetically related to Father 1 (the U.S. citizen) and Child 2 to Father 2; the U.S. Department of State only granted citizenship to Child 1. Under birthright citizenship laws, children born abroad to one American parent should qualify as citizens, so both children should have been granted citizenship. regardless of the DNA test results. The couple pursued legal action in the U.S. and after several years, a federal court ruled that Child 2 should have been granted citizenship at birth because the parents are legally married. This ruling was in contrast to the Assisted Reproductive Technology and Surrogacy Abroad policy by the

[1] M Flynn. One twin was a citizen, the other undocumented. A victory in court for their same-sex parents rebukes the State Department. *The Washington Post* (22 February 2019). Available at https://www.washingtonpost.com/nation/2019/02/22/one-twin-was-citizen-other-undocumented-victory-court-their-same-sex-parents-rebukes-state-dept/

AD Craythorne. Same-sex equality in immigration law: The case for birthright citizenship for foreign-born children of U.S. citizens in same-sex binational unions. *Texas Law Review* **97**, 645-671 (2019)

A Tchekmedyian. These twins were born 4 minutes apart. But only one is a U.S. citizen. *Los Angeles Times* (27 January 2018). Available at https://www.latimes.com/local/lanow/la-me-ln-twins-citizenship-20180127-story.html

	Department of State that specifies "a U.S. citizen father must be the genetic parent of the child and meet all other statutory requirements in order to transmit U.S. citizenship to the child at birth."[2]

Case 23: DNA documentation of a relationship is insufficient

**Source**	Study team reported
**Region**	North America
**DNA test type**	Relationship STR at a commercial AABB laboratory
**Relationship tested**	Siblings
**Interpreted value**	DNA testing can disprove or detect fraud when claimed relationships or identity are in question.
**Interpreted pitfall(s)**	Collection, processing, and comparison of DNA samples from multiple individuals can carry logistical, temporal, geographical, and financial burdens. The government might not collect samples or request or apply DNA testing results uniformly in decision-making processes.
**Narrative[3]**	A man born in the United States to two U.S. citizens was adopted by a family abroad. After the adoption, the last name on his birth certificate, which was originally both of his biological parents' last names, was changed to reflect both of his adoptive parents' last names. He grew up in the U.S. believing he was born abroad. He was once almost deported but won his case to remain. When immigration officials later attempted to deport him a second time, his adoptive mother told his wife that he was born in the U.S. and instructed her to help him. Fifteen (15) years prior, his adoptive family had found his biological brother's birth certificate; after a year and a half of searching, his adoptive family located not only his biological brother, but also five biological sisters. After USCIS gave him 30 days to prove his U.S. citizenship, his immigration attorney presented "DNA proof of the man's six biological siblings, medical records that prove he was born in a U.S. hospital to two American parents, a foreign birth certificate that indicates he was adopted, documents that show his original last name was changed and statements from his family, including a testament from his biological mother." Due to the changes in his last name, however, he is still in danger of being deported.

[2] U.S. Department of State. Assisted Reproductive Technology (ART) and surrogacy abroad. *U.S. Citizenship Laws and Policy* (2020). Available at https://travel.state.gov/content/travel/en/legal/travel-legal-considerations/us-citizenship/Assisted-Reproductive-Technology-ART-Surrogacy-Abroad.html

[3] M González. Despite being born in the US, adopted man says he is in danger of being deported to Mexico again. *NBC San Diego* (9 February 2021). Available at https://www.nbcsandiego.com/news/local/despite-being-born-in-the-us-adopted-man-says-he-is-in-danger-of-being-deported-to-mexico-again/2516803/

Case 24: Officials will not collect a DNA sample

**Source**	Study team reported
**Region**	West Africa
**Dna test type**	Relationship STR at a commercial AABB laboratory
**Relationship tested**	Parent-child
**Stated value**	DNA testing can provide documentation of genetic relationships and identity when other forms of documentation are unavailable, inaccurate, or insufficient to meet the burden of evidence.
**Stated pitfall(s)**	Collection, processing, and comparison of DNA samples from multiple individuals can carry logistical, temporal, geographical, and financial burdens. The government might not collect samples or request or apply DNA testing results uniformly in decision making processes.
**Narrative**[4]	A heterosexual married American couple is in the process of adopting two-year-old twins from a West African country. In 2018 they were living in the twins' country of origin; they were given custody of the children in the summer of 2018, and the adoption was finalized on the part of the West African country's government by the end of 2018. The couple began the U.S. adoption process at the beginning of 2019 and were invited to apply for adoption visas at the beginning of 2020 to return to the United States with their children. At their visa interview at the U.S. embassy located in a second West African country, however, immigration officials told the couple that they needed to provide more evidence. They began to gather materials, but border closures and travel restrictions due to the coronavirus pandemic made gathering all the required evidence impossible. Documents from the twins' country of origin are considered unreliable, so DNA evidence of the relationship between the birth parents and the twins would be helpful. The family now lives in the second West African country where their interview was held. One of the birth parents lives in the twins' country of origin; the U.S. embassy in the twin's country of origin will not cooperate to DNA test this birth parent. The family wishes to return to the United States due to safety concerns amid the pandemic, but cannot do so with the twins, who are not U.S. citizens. They, along with other families in similar positions, have petitioned the U.S. government to grant emergency exceptions for visas amid the pandemic.

[4] A Westerman. 'We'd Have to Abandon Our Daughters:' Pandemic Delays International Adoptions. *NPR* (14 April 2020). Available at https://www.npr.org/2020/04/14/831893954/we-d-have-to-abandon-our-daughters-pandemic-delays-international-adoptions

CONTEXT 4: DNA testing for verification of parent-child relationships at border entry points

Case 25: DNA testing request reveals fraud

**Source**	Study team reported
**Region**	Central America
**DNA test type**	Rapid DNA
**Relationship tested**	Father-child
**Interpreted value**	DNA testing requests or requirements can deter intentionally fraudulent misrepresentations of relationships or clarify misunderstandings around the kinds of relationships that qualify for immigration benefits.
**Interpreted pitfall(s)**	No pitfalls interpreted
**Narrative**[5]	A Central American man was encountered by Homeland Security Investigation agents in Texas. He was with an infant child that he initially claimed was his. While he was being interviewed for potential rapid DNA testing, he confessed that he had presented a fraudulent birth certificate for the infant child, who was not related to him. He had reportedly "purchased" the infant shortly after birth from the birth mother for the equivalent of approximately $84. The man did not present any documents to validate either a legal adoption of the infant or documents from the birth mother indicating her consent to legally transfer custody of the infant. The subject further admitted the infant was purchased solely as a means for him to enter the United States. The man was charged with violations of alien smuggling and illegal entry.

[5] Nevano, G.C. Statement of Gregory C. Nevano, Assistant Director, Investigative Programs, Homeland Security Investigations. U.S. Dpeartment of Homeland Security (26 June 2019). Available at https://www.hsgac.senate.gov/imo/media/doc/Testimony-Nevano-2019-06-26.pdf

CONTEXT 5: Comparison of family reference samples to unidentified human remains samples for identification purposes in transnational missing persons cases

Case 26: DNA testing documents a genetic relationship

**Source**	Study team reported
**Region**	Central America
**DNA test type**	STR typing at a forensic laboratory
**Relationship tested**	first-degree relative
**Interpreted value**	DNA testing can provide documentation of genetic relationships and identity when other forms of documentation are unavailable, inaccurate, or insufficient to meet the burden of evidence.
**Interpreted pitfall(s)**	No pitfalls interpreted
**Narrative**[6]	A woman left for the United States after her husband had been killed in her home country. She intended to join family in New York, find work, and send money back home. She crossed the U.S.-Mexico border in Texas; two days after she crossed, a deceased, unidentified woman was found in a county 50 miles north of the border. The remains of the woman were interred in a cemetery with a temporary marker that read "Unknown Female, [location where body was found]." Years later, a team of forensic anthropologists exhumed her remains as part of their efforts to identify transnational missing persons. They found among her remains, in the insoles of her shoes, an identification card bearing her name. This led the team to search NamUs for a corresponding missing persons' report. A report was found in NamUs, but no DNA sample had been obtained. Her family in her home country was contacted by an NGO who collected DNA samples from biological relatives. Two weeks later, a DNA association report, along with a comparison of antemortem and postmortem data, suggested that the remains did belong to the woman named on the ID card she was carrying.

Case 27: DNA testing documents a relationship, but is rejected by the family

**Source**	Study team
**Region**	Central America
**DNA test type**	STR typing at a forensic laboratory
**Relationship tested**	First-degree relative

[6] Rose, A. The forensics of identifying migrants who die exhausted after crossing from Mexico. in *Scientific American* (2015). Available at https://www.scientificamerican.com/article/the-forensics-of-identifying-migrants-who-die-exhausted-after-crossing-from-mexico/

**Interpreted value**	DNA testing can provide documentation of genetic relationships and identity when other forms of documentation are unavailable, inaccurate, or insufficient to meet the burden of evidence.
**Interpreted pitfall(s)**	Kinship analysis can reveal sensitive information. Collection, processing, and comparison of DNA samples from multiple individuals can carry logistical, temporal, geographical, and financial burdens. The government might not collect samples or request or apply DNA testing results uniformly in decision-making processes.
**Narrative**	Skeletal remains were found on private land in Texas. Near the remains, an identification card and passport with a name were discovered. Law enforcement notified the Justice of the Peace and the local funeral home buried the remains with a temporary marker using the name on the passport. However, no DNA specimen was submitted for genetic typing and comparison to a family reference sample prior to burial as required by state law. Furthermore, no official identification by a legal jurisdictional authority was performed; rather, an assumption was made that the remains were associated with the name on the passport. Because there had been no official identification, legally, the remains were classified as unidentified. Seven years after the remains were found, a team of forensic anthropologists exhumed them as part of their efforts to identify transnational missing persons. A DNA specimen was submitted to a government-funded laboratory for comparison to family reference samples collected by a consul. Notification of a genetic association was received almost a year later based on a partial STR profile. To date, the woman identified as the mother of the deceased via genetic association does not accept the identification as she believes she saw a photo of her child in a detention center after they were reported missing.

Case 28: Infrastructure delays or prevents a DNA identification

**Source**	Study team communication
**Region**	Central America
**DNA test type**	STR typing at a forensic laboratory and SNP typing at a commercial laboratory
**Relationship tested**	first-degree relative and distant kinship
**Interpreted value**	DNA testing can provide documentation of genetic relationships and identity when other forms of documentation are unavailable, inaccurate, or insufficient to meet the burden of evidence.
**Interpreted pitfall(s)**	Collection, processing, and comparison of DNA samples from multiple individuals can carry logistical, temporal, geographical, and financial burdens. DNA from the appropriate individuals to test a relationship is not always available. The appropriate technology and/or infrastructure to test a relationship is not always available.

**Narrative**	The body of an unidentified man was recovered and sent to a medical examiner for identification. The medical examiner estimated that the man was between 30-45 years old and had been deceased for several months at the time of recovery. Along with his remains was a scrap of paper with names and phone numbers and a photograph of a woman. Humanitarian groups have been unsuccessful investigating the phone numbers and photograph but found clues that the individual might be from a specific Central American country and that the photograph might be of the mother of his child. His remains were genotyped for STRs, but no kinship matches have resulted to date. It is possible that his family provided family reference samples to a database that is not being compared to the one that holds his data or that the family has not come forward to provide family reference samples. His DNA sample was also genotyped for SNP data in order to cross-reference his data with potential relatives' data in genealogical databases. Distant relatives were theorized from the data, but no identifications have been made to date. However, ancestry informative markers resulting from the SNP data provided more evidence that the he could be from the country identified by humanitarian groups.

Case 29: Infrastructure delays or prevents a DNA identification

**Source**	Study team communication
**Region**	Unknown
**DNA test type**	STR typing at a forensic laboratory
**Relationship tested**	First-degree relative
**Interpreted value**	DNA testing can provide documentation of genetic relationships and identity when other forms of documentation are unavailable, inaccurate, or insufficient to meet the burden of evidence.
**Interpreted pitfall(s)**	DNA from the appropriate individuals to test a relationship is not always available. The appropriate technology and/or infrastructure to test a relationship is not always available. The government might not collect samples or request or apply DNA testing results uniformly in decision-making processes.
**Narrative**[7]	As part of their efforts to identify transnational missing persons, a team of forensic anthropologists had plans to exhume at least eight unidentified individuals from eight burial sites in a cemetery based on the temporary grave markers that were still visible. They returned from the cemetery with 28 body bags, the majority from unmarked burial sites discovered by talking with local community members. One of the bodies was an unidentified individual who had been found on private land in Texas 5 years prior to exhumation. Contrary to state law, no DNA specimen had been collected prior to burial. A DNA

[7] Matthews, J.D. OP-ID. 32 minutes (2018). Available at https://filmfreeway.com/OP-ID

	specimen was submitted to a government-funded laboratory about a few months after exhumation for typing. Notification that a full profile was obtained and uploaded to the federal database known as the Combined DNA Index System [CODIS] was received approximately nine months later. To date, no family reference samples have matched. It is possible that the family of the deceased provided family reference samples to a database that is not being compared to the unidentified human remains index in CODIS or that the family has not come forward to provide family reference samples.

Case 30: Infrastructure delays or prevents a DNA identification

**Source**	Study team communication
**Region**	Unspecified
**DNA test type**	STR typing at a forensic laboratory
**Relationship tested**	First-degree relative
**Interpreted value**	No utility interpreted
**Interpreted pitfall(s)**	The appropriate technology and/or infrastructure to test a relationship is not always available. The government might not collect samples or request or apply DNA testing results uniformly in decision-making processes.
**Narrative**	A person crossed into Texas from Mexico with a group led by a coyote (a paid escort) over five years ago. The coyote escorting the group dropped them off at a location in Texas and told them to walk north to bypass a U.S. Customs and Border Protections checkpoint. Customs and Border Protection [CBP] agents spotted the group traveling, and a chase ensued that resulted in some migrants being captured and others scattering as they ran away. The person in question was among those who scattered and has not been heard from since. Periodic search and recovery efforts led by the county where the person disappeared and humanitarian groups have not yielded any sign of them. The families provided DNA family reference samples, assuming the worst has happened. Thus far, there have been no reports of a match to unidentified human remains. It is possible that the body has not been found or that the DNA data from the human remains has not been provided to a database for comparison to the data from the provided family reference samples.

Case 31: Officials will not collect a DNA sample

**Source**	Study team reported
**Region**	Unknown
**DNA test type**	STR typing at a forensic laboratory
**Relationship tested**	First-degree relative

**Interpreted value**	No utility interpreted
**Interpreted pitfall(s)**	The government might not collect samples or request or apply DNA testing results uniformly in decision-making processes.
**Narrative**[8]	During the coronavirus pandemic, Border Patrol agents found a person who was dehydrated and semi-responsive in Texas. The person was taken to a Texas hospital where they were sedated and placed on a ventilator. They tested positive for COVID-19 and died a few days later. They were not carrying any identification and CBP did not find a match for their fingerprints in their system. The person's remains could not legally be buried or cremated prior to DNA sampling, but the hospital would not collect DNA samples without an autopsy and would not preform autopsies on COVID-positive patients. The remains were moved to a separate, refrigerated trailer while authorities determined how to identify the person so the family could be notified and the remains returned to them. No further information on the case has been reported.

[8] Gonzalez, V. Death of unidentified migrant who died in federal custody after testing positive for COVID-19 highlights complex state process. *KRGV.com* (10 July 2020). Available at https://www.krgv.com/news/death-of-unidentified-migrant-who-died-in-federal-custody-after-testing-positive-for-covid-19-highlights-complex-state-process/
